# Paired metabolomics and volatilomics provides insight into transient high light stress response mechanisms of the coral *Montipora mollis*

**DOI:** 10.1007/s11306-024-02136-9

**Published:** 2024-06-17

**Authors:** Natasha Bartels, Jennifer L. Matthews, Caitlin A. Lawson, Malcolm Possell, David J. Hughes, Jean-Baptiste Raina, David J. Suggett

**Affiliations:** 1 Climate Change Cluster, Faculty of Science, University of Technology Sydney, Ultimo, NSW Australia; 2 Heron Island Research Station, Faculty of Science, University of Queensland, Gladstone, 4680 Australia; 3 School of Life and Environmental Sciences, University of Sydney, Sydney, NSW Australia; 4 National Sea Simulator, Australian Institute of Marine Science, Townsville, QLD Australia; 5 KAUST Reefscape Restoration Initiative (KRRI) and Red Sea Research Center (RSRC), King Abdullah University of Science and Technology, Thuwal, Saudi Arabia

**Keywords:** Coral, Metabolomics, Volatilomics, Light-stress, Holobiont

## Abstract

The coral holobiont is underpinned by complex metabolic exchanges between different symbiotic partners, which are impacted by environmental stressors. The chemical diversity of the compounds produced by the holobiont is high and includes primary and secondary metabolites, as well as volatiles. However, metabolites and volatiles have only been characterised in isolation so far. Here, we applied a paired metabolomic-volatilomic approach to characterise holistically the chemical response of the holobiont under stress. *Montipora mollis* fragments were subjected to high-light stress (8-fold higher than the controls) for 30 min. Photosystem II (PSII) photochemical efficiency values were 7-fold higher in control versus treatment corals immediately following high-light exposure, but returned to pre-stress levels after 30 min of recovery. Under high-light stress, we identified an increase in carbohydrates (> 5-fold increase in arabinose and fructose) and saturated fatty acids (7-fold increase in myristic and oleic acid), together with a decrease in fatty acid derivatives in both metabolites and volatiles (e.g., 80% decrease in oleamide and nonanal), and other antioxidants (~ 85% decrease in sorbitol and galactitol). These changes suggest short-term light stress induces oxidative stress. Correlation analysis between volatiles and metabolites identified positive links between sorbitol, galactitol, six other metabolites and 11 volatiles, with four of these compounds previously identified as antioxidants. This suggests that these 19 compounds may be related and share similar functions. Taken together, our findings demonstrate how paired metabolomics-volatilomics may illuminate broader metabolic shifts occurring under stress and identify linkages between uncharacterised compounds to putatively determine their functions.

**Supplementary Information:**

The online version contains supplementary material available at 10.1007/s11306-024-02136-9.

## Introduction

Maintenance of endosymbiotic algae (Family: Symbiodiniaceae, LaJeunesse et al., 2018) in corals is underpinned by effective metabolic compatibility between the partners (Davy et al., 2012; Matthews et al., 2017; Nitschke et al., 2022; Suggett et al., 2017), and relies on availability of light for photosynthesis (Kleypas et al., 1999; López-Londoño et al., 2021). Light fuels photosynthesis of Symbiodiniaceae, which in turn translocate metabolites to the coral host, such as carbohydrates, amino acids, and fatty acids (Burriesci et al., 2012; Hillyer et al., 2017, 2018; Kopp et al., 2015; Matthews et al., 2020; Papina et al., 2007). In return, inorganic carbon, nitrogen and other necessary substrates for photosynthesis and growth are translocated to the endosymbionts (Davy et al., 2012; Morris et al., 2019).

Light intensity and quality both greatly affect the ecological success of reef-building corals but are highly dynamic spatiotemporally (Ben-Zvi et al., 2021; Mass et al., 2010; Roth, 2014). Variability of light intensity in shallow reef environments is particularly pronounced (Lohr et al., 2019; Roth, 2014), with transient high light exposure occurring on scales of seconds via cloud-induced light flecking (Perkins et al., 2006) and wave lensing (Banaszak & Lesser, 2009; Veal et al., 2010). Shifts in light also occur over larger time-scales, such as weeks or months from seasonal variation in incident light (Vajed Samiei et al., 2016; van Hoytema et al., 2016). Excessive light exposure can be harmful for corals, as high intensities elevate oxygen partial pressures and increases reactive oxygen species (ROS). Production of ROS that overwhelms antioxidant capacities in turn drives oxidative stress, which may play a role in coral bleaching (i.e., the loss of Symbiodiniaceae from coral tissues) (Suggett & Smith, 2020), including damage to DNA resulting in impaired fitness (Downs et al., 2013). High light intensities can also result in greater susceptibility to disease (Rosenberg & Loya, 2013) and abiotic stressors such as increased sea surface temperatures (Hawkins et al., 2015; Rosic et al., 2020). Photophysiological responses and adaptations to light availability have been widely studied in corals (e.g., Cohen & Dubinsky, 2015; DiPerna et al., 2018; Juhi et al., 2021; Suggett et al. 2022; Nitschke et al., 2022). Corals have evolved physiological, morphological, and behavioural mechanisms to fine-tune light exposure and optimise the performance of their photosynthetic Symbiodiniaceae as well as photophysiological adjustments by the Symbiodiniaceae themselves (Lohr et al., 2019; Nitschke et al., 2022; Roth, 2014). Such fine-tuning ultimately affects the production and translocation of metabolites from Symbiodiniaceae to their hosts, as well as the metabolic requirements and response of the coral holobiont. However, major gaps remain in our understanding of how these organisms biochemically respond to transient light stress.

Metabolomic approaches have identified compounds and compound classes – for example – glucose (e.g., Burriesci et al., 2012), docosahexaenoic acid (e.g., Hillyer et al., 2017), glycans and lectins (e.g., Markell & Wood-Charlson, 2010), and inositol (e.g., Matthews et al., 2017) that may be key in regulating the coral-Symbiodiniaceae symbiosis (Rosset et al., 2021). Metabolomics has also proven to be a valuable tool for characterising the metabolic mechanisms underlying the coral-dinoflagellate symbiosis maintenance or dysfunction under stress (Haydon et al., 2023; Hillyer et al., 2017; Matthews et al., 2017). For example, Hillyer et al. (2018) detected a 13.5-fold increase in inositol and a 12-fold increase in glucose in coral host tissues under heat stress, as well as an increase in fatty acids C12:0 and C14:0 (~ 5-fold and ~ 3-fold respectively), suggesting changes to the catabolism of lipid pools. Such findings indicate increased energetic demands across the holobiont during thermal acclimation. Light-driven metabolic changes in corals can induce changes in specific metabolite groups such as UV-absorbing mycosporine-like amino acids (Rosic & Dove, 2011; Torres et al., 2007) and fatty acids (Treignier et al., 2008), or the antioxidant dimethylsulfoniopropionate (DMSP; Deschaseaux et al., 2014; Yost et al., 2012). Untargeted metabolomics has further revealed a metabolic shift likely occurs in corals under high irradiance prior to measurable photophysiological responses (Lohr et al., 2019) including an increase in amino acids, fatty acids (also Treignier et al., 2008) and sugar alcohols, and a decrease in campesterol. Metabolic shifts may therefore maintain oxidative state and osmolality of the host, as well as increased turnover of lipid stores and reduced translocation of photosynthetic products from symbiont to host (Lohr et al., 2019). While these studies begin to highlight the link between photoacclimation and metabolism, due to the chemical diversity of metabolites, multiple metabolomic approaches are required to reveal the full diversity of metabolites produced by corals and their symbionts (Lawson et al., 2022).

Corals emit a large range of volatile molecules, with different components of the coral holobiont (e.g., the host, Symbiodiniaceae and associated bacteria) contributing to the production of these complex and species-specific biogenic volatile organic compounds (BVOCs – gaseous metabolites; Lawson et al., 2019; Olander et al., 2021, 2023). Volatiles emitted from corals may play various roles in stress response, chemical signalling, and may have antimicrobial activity (Lawson et al., 2020). The volatilome can also shift under changing environmental conditions with experimental heat stress studies identifying dramatic losses of volatile diversity and richness in both the holobiont and Symbiodiniaceae (Lawson et al., 2019, 2020). Comparatively to primary and secondary metabolites, few studies have investigated changes in specific volatiles under light stress (e.g., dimethyl sulfide; Deschaseaux et al., 2014). While the power gained by pairing these approaches has been utilised across other fields – for example, in food chemistry to detect metabolic markers of certain flavours (Li et al., 2023), or in medicine for early cancer detection using urine (Mallafré-Muro et al., 2021) – no studies to date have paired metabolomics and volatilomics to study coral holobiont stress. Combining volatilomics and metabolomics may provide greater understanding of the metabolic processes underlying photoacclimation and high-light stress responses in the coral holobiont. Here, we identified shifts in the metabolism of the coral *Montipora mollis* under transient light stress using a paired volatilomic and metabolomic approach. This multi-omics approach could help to identify new stress-related compounds and improve our understanding of coral metabolism under light stress.

## Materials and methods

### Coral collection and growth conditions

Four colonies of *Montipora mollis* (green colour-morph) were sourced from Batavia Coral Farms (Geraldton, WA), originally collected from a reef off the Abrohlos Islands (Western Australia; 28°52’43.3"S 114°00’17.0"E) at a depth of 1 m. *M. mollis* was selected as the metabolome of *Montipora* species has previously been characterised under heat stress, but not under light stress. Coral colonies were held under blue and white light at 185 µmol photons m^− 2^ s^− 1^ and at 25 °C for three months before shipment to the University of Technology Sydney (UTS). Upon arrival at UTS, corals were maintained in an 800 L aquarium (University of Technology Sydney) containing artificial seawater maintained at 35 PSU (Reef Crystals, Instant Ocean, Aquarium Systems, Wicklife, OH, USA), measured daily using a digital refractometer (HI 96,800, Hanna Instruments, Woonsocket, RI, USA). Light was delivered on a 12:12 h light: dark cycle by three LED lights (Hydra 52, AquaIllumination, Ames, IA, USA) with a daily maximum intensity of ~ 150 µmol photons m^− 2^ s^− 1^ of blue-white light as measured directly above the colony surface (See Hughes et al., 2022); maintaining a slightly reduced light level can improve the health of newly introduced corals in aquaculture, as well as minimising competition from crustose coralline algae and macroalgae (Ramsby et al., 2024). Temperature was maintained at 25 ± 0.5 °C (checked daily). Alkalinity, calcium, and magnesium were maintained at 125 ± 3ppm, 450 ± 10 ppm and 1350 ± 50 ppm respectively, measured weekly using a commercial test kit (Reef Foundation Pro, Red Sea, Houston, TX, USA). Nitrate (NO^3−^) and phosphate (PO_4_^3−^) levels were maintained at or below 4 ppm and 0.01 ppm respectively, measured fortnightly with a commercial test kit (Algae Control Pro, Red Sea, USA). To maintain optimal water chemistry and quality for coral growth, a water change of ~ 10% of the total aquarium volume was performed weekly. Continuous aeration was provided by the main return pump, a gyre generator (XF250, Maxspect, Hong Kong, China) and a venturi-type protein skimmer rated for 1500 L (unbranded). Following one month of recovery from shipping and acclimation, colonies were divided into fragments with an approximate surface area of 25 cm^2^ of photosynthetically active tissue, using a Dremel tool equipped with a diamond point circular saw blade (Dremel, Racine, WI, USA). Corals were then left for a further six weeks of recovery before experiments began, ensuring tissue extension over skeleton exposed by cutting.

### Transient high-light stress event

Coral fragments were placed upon a coarse mesh platform in air-tight, 500 mL acrylic chambers containing 300 mL of aquarium water. Chambers were maintained at an optimal temperature (25 ± 0.5 °C) under LED lights (Hydra 52, AquaIllumination, Ames, IA, USA) set to each treatment intensity. Specifically, fragments were subjected to either their control light intensity (~ 150 µmol photons m^− 2^ s^− 1^; *n* = 6), or were exposed to higher irradiance (~ 1200 µmol photons m^− 2^ s^− 1^; *n* = 6) for 30 min. Volatile compounds of each fragment were measured using headspace sampling (as described below), run sequentially on two replicates at a time under their respective light treatment. Control and treatment fragments were alternately run (two control replicates, followed by two treatment replicates and so on until all fragments had undergone their respective light treatment) to account for any potential diel effects throughout the day of sampling. Fragments were immediately snap frozen after each run and stored at -80 °C for metabolomic analysis.

### Active fluorometry

Photochemical efficiency of six additional fragments (*n* = 3 under control conditions, *n* = 3 under treatment conditions) were examined in parallel to the incubation experiments, using LIFT Fast-Repetition Rate fluorometry (following Suggett et al. 2022). Flashlets of blue light (470 nm peak wavelength) were delivered to samples using an excitation protocol with a saturation sequence followed by a relaxation sequence. Saturation consisted of 300 flashlets of 1.6 µs duration at fixed intervals of 2.5 µs, whereas relaxation consisted of 127 flashlets of 1.6 µs duration with an initial interval of 20 µs, followed by exponentially decreasing intervals by a factor of 1.025. A kinetics script was executed to determine the (1) maximum PSII photochemical efficiency (*F*_v_*/F*_m_) after 15 min low light (~ 10 µmol photons m^− 2^ s^− 1^) acclimation; (2) PSII operating efficiency (*F*_q_´/*F*_m_´) after 30 min at treatment (1200 µmol photons m^− 2^ s^− 1^) or control (150 µmol photons m^− 2^ s^− 1^) irradiance; and (3) *F*_v_*/F*_m_ following a 30 min recovery period under low light (10 µmol photons m^− 2^ s^− 1^). The above protocol was run for 14 induction-relaxation sequences (each consisting of 1 flash, equal to 10 flashlets) in darkness, followed by a further 140 induction-relaxation sequences under blue light (445–470 nm) at 1200 µmol photons m^− 2^ s^− 1^ for treatment samples and 150 µmol photons m^− 2^ s^− 1^ for control samples. The recovery period consisted of a further 140 induction-relaxation sequences under 10 µmol photons m^− 2^ s^− 1^ of light.

### Volatile compound sampling and analysis

Fragments in the chambers were subjected to their respective treatment irradiances, and volatile sampling was performed using the methods outlined in Lawson et al. (2020). Each chamber was purged for 30 min at 100 mL/min using instrument grade air (BOC Gases, Linde Group) and the outlet collected during the last 20 min of purge time on thermal desorption (TD) tubes (200 mg Tenax TA; Markes International Ltd), which were immediately capped and stored at 4 °C until analysis. Following the purge, coral fragments were snap frozen in liquid nitrogen and stored at -80 °C for metabolite analysis. The sampling process was repeated for blanks containing only aquarium seawater under control and treatment conditions (*n* = 3 per treatment group).

TD tubes were processed as described in Lawson et al. (2020). Prior to desorption, 0.2 µL of Chlorobenzene-D5 was added to each tube as an internal standard. TD tubes were desorbed at 300 °C for 6 min using an automated thermal desorption unit, then concentrated at -30 °C on a Tenax TA cold trap, followed by flash heating to 300 °C and injection via a transfer line held at 150 °C onto a 7890 A GC-MS (Agilent Technologies Pty Ltd). A 60 m × 0.32 mm, 1 μm film thickness BP1 capillary column (SGE Analytical Science Pty Ltd) was fitted to the GC-MS, run splitless at a flow rate of 2.3 mL min^− 1^. The GC oven was heated to 35 °C for 5 min, followed by 4 °C min^− 1^ to 160 °C, then 20 °C min^− 1^ to 300 °C and held for 10 min to allow for complete desorption. A mass-selective detector (Model 5975 C; Agilent Technologies Pty Ltd) was coupled to the GC-MS, set to a scanning range of 35–250 amu.

Common contaminating ions (73, 84, 147, 149, 207 and 221 m/z) were removed using the denoising function in OpenChrom Lablicate Edition (version 1.5.0, Lablicate GmbH, Germany). Further pre-processing was performed using the metaMS package (Wehrens et al., 2014) through the Galaxy Project platform (The Galaxy Community, 2022). Compounds were automatically assigned to peaks using NIST Mass Spectral Search Program (NIST MS Search 2.2; NIST, Gaithersburg, Maryland, USA), then manually verified to ensure mass spectra correctly matched the assigned compound. Compound peak areas were then normalised to the peak area of the internal standard, and the average values for compounds detected in the seawater blanks subtracted from all samples. If values were negative after blank substractions (due to detection in samples at lower abundances than in blanks), they were considered equal to 0. Finally, all values were normalised to the surface area of the coral, determined using the single wax dipping method (Veal et al., 2010; Grottoli et al., 2021).

### Metabolite sampling

Metabolite extractions (used hereafter to refer to metabolites that were not sampled via headspace sampling onto TD tubes) were performed on the holobiont as per Matthews et al. (2023). Tissue was removed from the previously frozen coral skeletons via airbrushing into 10 mL of Milli-Q water chilled to 4 °C. This tissue slurry was then lyophilized to complete dryness overnight in an Alpha 2-4LD freeze dryer (CHRIST, Germany). Metabolites were extracted from 25 mg of dried tissue by adding 400 µL of 100% cold (-20 °C) LC-MS grade methanol with 20 µg mL^− 1^ final concentration internal standard (D-Sorbitol-^13^C_6_) then bead milling at 50 Hz for 3 min (710–1180 μm acid-washed glass beads, Sigma-Aldrich, United States). An additional 600 µL of 100% cold (-20 °C) methanol (with internal standard) was added, then samples were vigorously vortexed and placed on a rotary shaker at 4 °C for 30 min. Samples were centrifuged at 3000 × g for 30 min at 4 °C, and the supernatant (metabolite extract) was transferred to a new tube. The remaining pellet was washed with 1000 µL of 50% cold (4 °C) methanol, vortexed for 1 min, and centrifuged again at 3000 × g for 30 min at 4 °C. The supernatant was pooled with the first extracts, then stored at -80 °C until analysis. The pellet was also stored at -80 °C for analysis of protein content. Samples where then centrifuged at 16,000 × g for 15 min to remove precipitates and 250 µL of each sample were dried down in 50 µL increments in glass inserts using an Eppendorf Vacufuge 5301 Concentrator Centrifuge (Eppendorf, Germany).

Metabolite samples were processed and derivatised online via GC-MS by *Metabolomics Australia* (University of Melbourne) using a Shimadzu AOC6000 autosampler, 2030 Shimadzu gas chromatograph (GC) and a TQ8040 triple-quadrupole mass spectrometer (MS; Shimadzu, Japan). Dried-down samples were reconstituted in 25 µL of Methoxyamine Hydrochloride (30 mg mL^− 1^ in Pyridine), then shaken at 37 °C for 2 h. A 25 µL aliquot of *N*,*O*-*bis* (Trimethylsilyl)trifluoroacetamide with Trimethylchlorosilane (BSTFA with 1% TMCS, Thermo Scientific) was then added to each sample, and incubated for 1 h at 37 °C. 1 µL of sample was injected onto the GC column (30 m Agilent DB-5 column with 1 μm film thickness and 0.25 mm internal diameter column) using a hot needle technique, with a 1:10 split. Helium with a flow rate of 1 mL min^− 1^ was used as the carrier gas, and Argon as the collision cell gas to generate the Multiple Reaction Monitoring (MRM) product ion. The injection temperature was set at 280 °C and the ion source to 200 °C. Analysis temperatures were programmed starting with 100 °C held for 4 min, followed by an oven temperature ramp of 10 °C min^− 1^ to 320 °C, held for 11 min. Approximately 520 quantifying MRM targets were collected using Shimadzu Smart Database along with qualifier ions for each target, which covers about 350 endogenous metabolites and multiple ^13^C labelled internal standards. Chromatograms and MRMs were assessed in the Shimadzu GC-MS browser and LabSolutions Insight software. Data were normalised to the relative abundance of the internal standard, and then to the protein content of the remaining tissue that the metabolites were extracted from, calculated using the Bradford Colorimetric method (Bradford, 1976) as described in Matthews et al. (2023) and adapted from Smart et al. (2010). Different normalisations were used for volatile metabolites (to surface area) and metabolites (to protein content of the extracted tissue), as the volatiles were measured from the entire coral fragment, and metabolites from a sub-fragment. As the concentrations of metabolites versus volatile metabolites are on vastly different scales, no comparisons of abundance are performed between them (see below) - thus these different normalisation techniques do not affect our analysis.

### Statistical analysis

Values of PSII photochemical efficiency between control and treatment groups were compared prior to the high-light treatment, directly following 30 min under high light, and after 30 min of recovery using an analysis of variance (ANOVA; with treatment group and timepoint as factors) and Tukey’s HSD pairwise comparisons.

While volatilomics via GC-MS has the benefit of high coverage, it also affected by high spectral convolution that can create difficulty in assigning peaks with certainty to known compounds (Yuan et al., 2022). We therefore remove volatile compounds from the analysis if they appeared in three or less replicates per treatment groups (original *n* = 6 per treatment group) to lessen noise within treatment groups (similar thresholding has been applied previously e.g., Lawson et al., 2020). This decision was made in order to draw conclusions with the greatest confidence for this initial application of integrated metabolomic and volatilomic data, and aligns with recommendations on minimum sample size proposed by the Metabolomics Standards Initiative (Sumner et al., 2007). Metabolites were excluded where the residual standard deviation was > 50% across replicates within treatment groups, as noise from individual variance can mask broader trends across treatments (Pang et al., 2021). As described above, different thresholding for volatile and metabolite data does not affect our analysis as no comparisons of abundance are made between datasets. Both datasets were log_10_-transformed and mean-centred prior to analysis. Principal Component Analyses (PCAs) and t-tests were used to compare differences in abundance of every compound between control and high-light groups, for both volatile and metabolite data separately using MetaboAnalyst 5.0 (Pang et al., 2021). Permutational multivariate analysis of variance (PERMANOVA) was performed on Euclidean distances with 999 permutations using the Vegan package (version 2.6.4) in R (version 4.2.1; R Core Team, 2022) to determine if the volatilome and metabolome of control versus high-light corals differed significantly. Spearman Rank Correlation with Holm p-value adjustments was performed on the combined volatilome and metabolome datasets using the Psych package (version 2.3.6). To determine whether this correlation analysis could be used to tentatively map volatiles to metabolic pathways, we first determined if metabolites that were significantly correlated (> 0.7 or < -0.7 and p-value < 0.05) belonged to the same Kyoto Encyclopedia of Genes and Genomes (KEGG; Kanehisa & Goto, 2000) pathway. This was true for 45% of correlated compounds (Table S1). We therefore selected all compounds (both volatiles and metabolites) that were significantly different between control and treatment groups (t-test, adjusted *p* < 0.05), visualised the correlations, and mapped volatiles to potential metabolic pathways (obtained from the KEGG database) via their correlations to metabolites. An additional network analysis with significant positive correlations between all compounds, including those that did not differ significantly between control and treatment, was also performed in Cytoscape 3.9.1 (Shannon et al., 2003). Compounds were arranged into clusters if each compound correlated to at least 5 other compounds in the cluster.

## Results

### Photophysiology

**Fig. 1:**
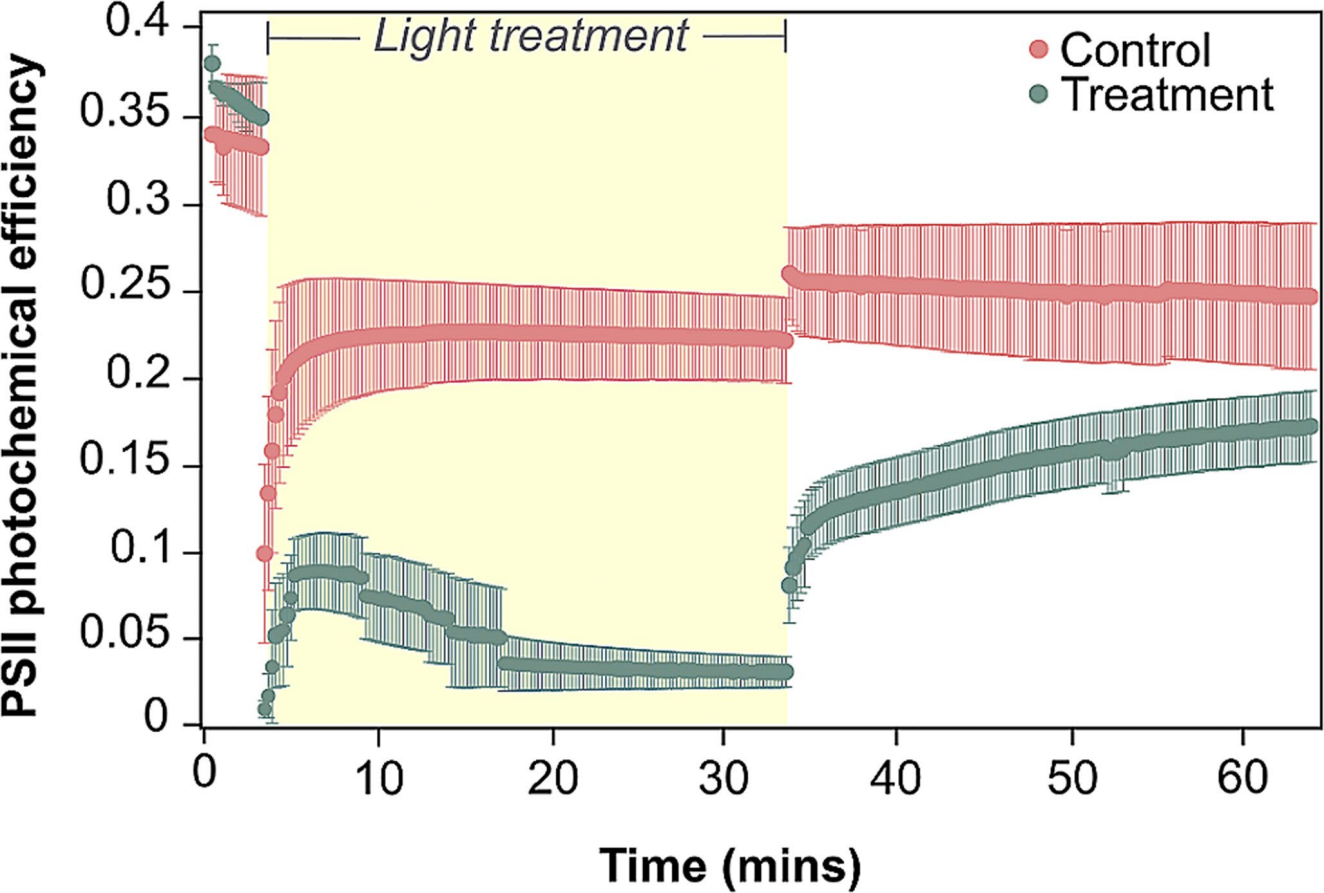
Photosystem II (PSII) photochemical efficiency of corals subjected to high-light (green) versus control conditions (pink). Yellow shading represents experimental light exposure; 150 µmol photons m^− 2^ s^− 1^ for control fragments, and 1200 µmol photons m^− 2^ s^− 1^ for treatment fragments. Measurements were performed before the transient exposure to high-light event, at the end of high-light exposure, and after 30 min of recovery (under 10 µmol photons m^− 2^ s^− 1^). A significant difference was only observed directly following 30 min of high-light. Error bars represent standard error of the mean (*n* = 3)

A significant interaction was identified between timepoints and treatment groups (ANOVA, *Df* = 2, *F* = 9.058, *p* = 0.004). Initial values of PSII photochemical efficiency did not differ between control (0.340 ± 0.038 = mean ± standard error of the mean hereafter) and treatment (0.381 ± 0.010) groups before the high light treatment was applied (Tukey’s HSD, *p* = 0.888; Fig. 1; Table S2). Following 30 min under high light, PSII photochemical efficiency was seven times lower for treatment corals (0.031 ± 0.009) compared to controls (0.222 ± 0.025; *p* = 0.003). However, there was no significant difference in photochemical efficiency between treatments after 30 min of recovery (control = 0.247 ± 0.042, treatment = 0.173 ± 0.020; *p* = 0.425). As such, the light stress used here was sufficient to elicit a transient light stress response that did not induce prolonged photodamage.

### Volatile compounds

**Fig. 2:**
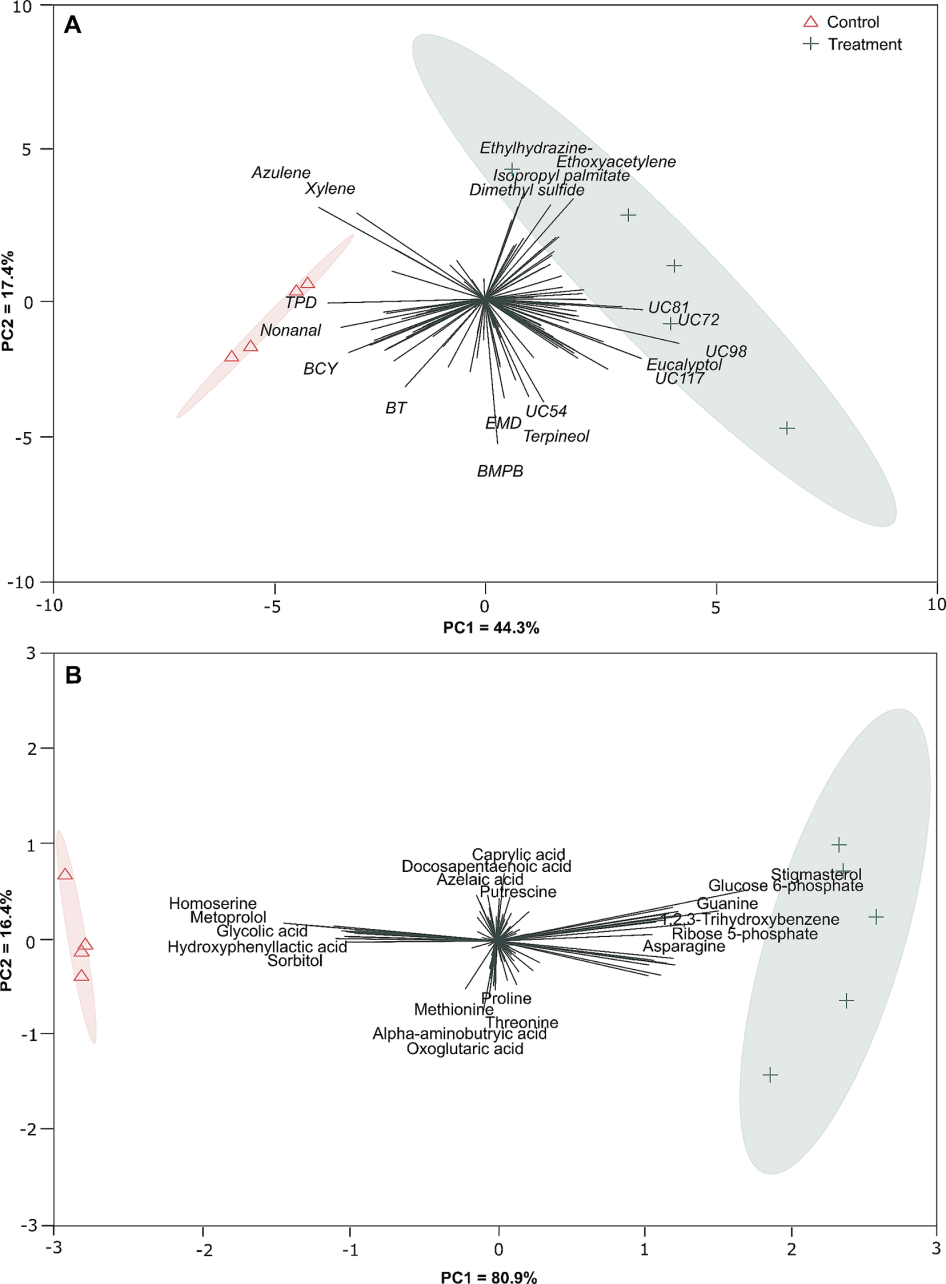
Principal Component Analysis (PCA) displaying differences between compounds produced by corals following different light exposure. (**A**) Differences in volatiles between light treatments, where the sum of the first two components = 61.7% of the variance. (**B**) Differences in metabolites between light treatments, where the sum of the first two components = 97.3% of the variance. Specific compound names have been shortened for clarity: TPD = 2,2,4 trimethy-1,3-pentanediol diisobutyrate; BCY = benzene, (2,4-cyclopentadien-1-ylidenemethyl)-; BT = 1,1’-biphenyl,2,2’5,5’-tetramethyl-; EMD = 2-ethyl-5-methyl-3,3-diphenylpyrrolidine; BMPB = benzene, 1,1’-(1-methyl-1,3-propanediyl)bis; UC = Unknown compound

**Fig. 3:**
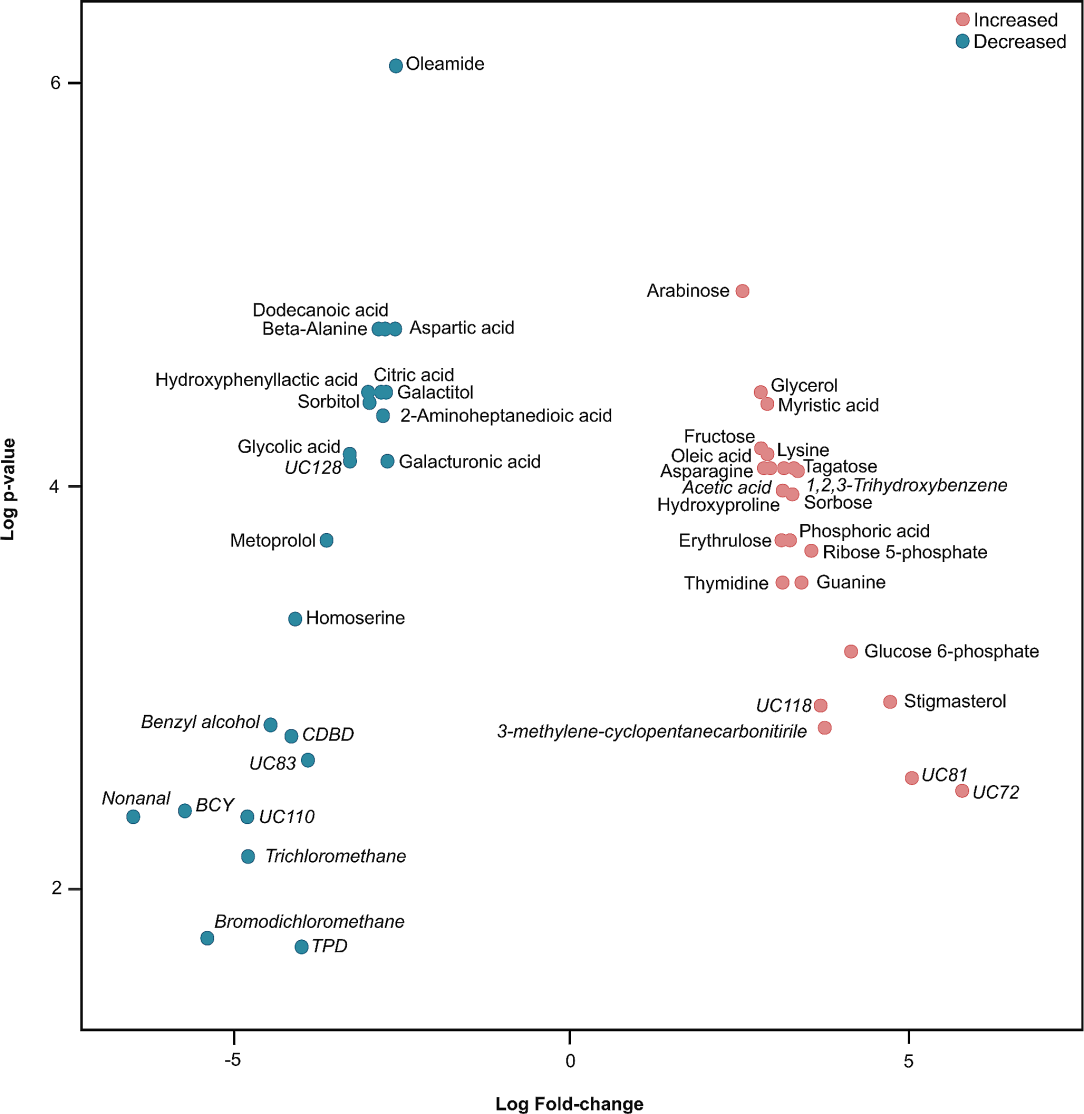
Log fold change of compounds (volatiles are italicised, metabolites are plain font) between control and high-light treatments. Red points indicate compounds that experienced a greater than two-fold increase in abundance, blue points indicate a greater than two-fold decrease. Specific compound names have been shortened for clarity: BCY = benzene, (2,4-cyclopentadien-1-ylidenemethyl)-; CDBD = 2,5-cyclohexadiene-1,4-dione, 2,6-bis(1,1-dimethylethyl); TPD = 2,2,4 trimethy-1,3-pentanediol diisobutyrate; UC = Unknown compound

A total of 113 volatile compounds were recorded following data normalisation and quality control, with 67 recorded in the control group and 89 under high-light stress. 26 of these compounds (23% of total volatiles found) could not be identified against the NIST MS Search compound libraries. High-light versus control groups showed clear clustering (PCA, Fig. 2A), with separation between the two conditions (PERMANOVA, *F*(1, 7) = 3.498, *R*² = 0.333, *p* = 0.013; Fig. S1) largely driven by benzene, 1,1’-(1-methyl-1,3-propanediyl)bis-, terpineol, ethyl-hydrazine, azulene and an unknown compound (UC54; PC loadings > 0.2 or < -0.2; Table S3). In total, 10 compounds significantly decreased in abundance under high-light treatment compared to controls (Fig. 3; Table S5). Notably, nonanal, benzene, (2,4-cyclopentadien-1-ylidenemethyl)-, bromodichloromethane, trichloromethane, benzyl alcohol and an unknown volatile compound (UC110) underwent a > 95% decrease in abundance in the high-light treatment. Six volatiles (acetic acid, 1,2,3-trihydroxybenzene, cyclopentanecarbonitrile, 3-methylene- and three unknown compounds) increased in abundance by > 10-fold under high light, with one unknown compound (UC72) increasing 55-fold.

### Metabolites

Following data normalisation and quality control, a total of 121 compounds were recorded remained, with 101 in the control group, and 108 in the high-light group. Similar to the volatile data, high-light versus control groups showed distinct clustering (PCA, Fig. 2B), with separation between groups (PERMANOVA, *F*(1, 7) = 3.066, *R*² = 0.305, *p* = 0.013; Fig. S1), driven by stigmasterol, glucose 6-phosphate, ribose 5-phosphate and homoserine (PC loadings > 0.2 or < -0.2; Table S4). A total of 31 compounds were significantly different between the fragments exposed to high-light and the controls (Fig. 3; Table S6). 17 compounds increased in abundance by > 5-fold under high-light stress relative to the controls, with the greatest increases observed in stigmasterol (27-fold), glucose 6-phosphate (18-fold), ribose 5-phosphate (12-fold) and guanine (11-fold). Comparatively, 13 compounds decreased in abundance by > 80%, with > 90% decreases observed in metoprolol and homoserine.

### Correlation and network analysis

**Fig. 4:**
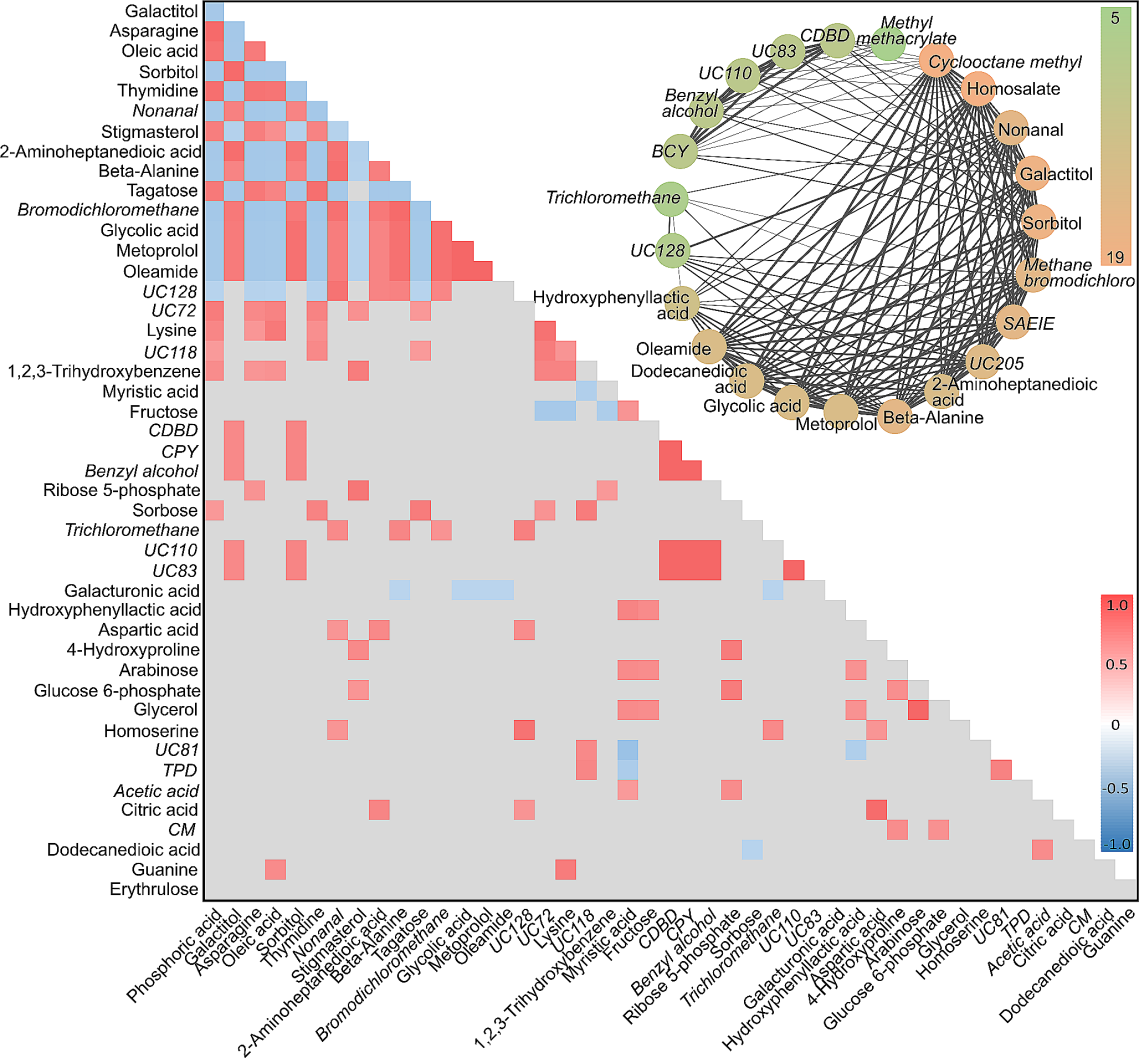
Heatmap displaying correlation coefficients between compounds produced by Spearman Rank correlation. Coloured values indicate a Holm-adjusted *p*-value of < 0.05, while grey shading indicates non-significance (*p* > 0.05). Red indicates a positive correlation, while blue indicates a negative correlation. Volatile compounds are italicised, and metabolites are in plain text. The circle of compounds displayed here are a subset of the full network analysis performed (see Fig. S2), depicting high numbers of correlations between all compounds in the circle (with each compound positively correlated to at least five other compounds in the circle). The green-orange colour scale represents the number of correlations a compound has, ranging from 5 to 19. The boldness of the lines indicates the strength of the correlation, from thinnest to thickest representing correlations between 0.7 and 1. Specific compound names have been shortened for clarity: TPD = 2,2,4 trimethy-1,3-pentanediol diisobutryate; BCY = benzene, (2,4-cyclopentadien-1-ylidenemethyl)-; BT = 1,1’-biphenyl,2,2’5,5’-tetramethyl-; CDBD = 2,5-cyclohexadiene-1,4-dione, 2,6-bis(1,1-dimethylethyl); EMD = 2-ethyl-5-methyl-3,3-diphenylpyrrolidine; BMPB = benzene, 1,1’-(1-methyl-1,3-propanediyl)bis; UC = unknown compound

Only ~ 12% of the volatiles that were significantly different between the light treatments were assigned to metabolic pathways in KEGG, and we therefore investigated potential correlations between the abundance of these molecules with the aim to link these volatiles to specific metabolic pathways. This approach was tested first on non-volatile metabolites and determined that 45% of the compounds significantly correlated (*r* > 0.7 or *r* < -0.7, and *p-*value < 0.05) belonged to the same KEGG pathway. Spearman rank correlation was then performed solely on the compounds that were significantly different between high light and control (adjusted *p*-value < 0.05; Fig. 4, Table S5), resulting in 143 positive and 72 negative correlations that were statistically supported (Fig. 4). All volatile compounds were found to be correlated to at least two metabolites. Five of these volatile compounds were positively correlated to two metabolites, sorbitol and galactitol. More broadly, trends between compound classes were also observed. For example, increases in sugars correlated to increases in fatty acids (e.g., a > 5-fold increase in arabinose and fructose correlated to a 7-fold increase in myristic acid). Fatty acids were also found to be negatively correlated to fatty acid derivatives (e.g., a 7-fold increase in oleic acid versus a > 80% decrease in both nonanal and oleamide).

A network was created for all positive correlations (*r* > 0.7, *p* < 0.05), with clusters assigned based on whether compounds correlated to at least five other compounds in the cluster; this was the lowest threshold at which compounds were observed to naturally cluster into groups (see Fig. S1 for full network). Notably, a cluster containing 23 compounds was observed, with sorbitol, galactitol, cyclooctane methyl and homosalate, accounting for the greatest number of correlations in the cluster (> 18 correlations each; Fig. 4). Sorbitol and galactitol are both found on the pentose phosphate and fructose metabolism pathways, and correlated to 18 other compounds in the cluster (including to each other); 11 volatiles and six metabolites. Of these compounds, 18 underwent a > 80% decrease in abundance under high-light. Three volatiles – nonanal, benzene, (2,4-cyclopentadien-1-ylidenemethyl)- and bromodichloromethane – decreased by > 97% under transient light stress.

## Discussion

The impacts of various stressors on coral metabolism have been well documented using traditional metabolite extraction methods (Matthews et al., 2023; Lawson et al., 2022). However, given the diversity of chemicals that can be produced by the coral holobiont, traditional metabolomics alone cannot fully characterise the chemical interplay occuring under stress. Here for the first time, we applied a multi-omics approach to better characterise coral holobiont metabolism, observing that transient high light stress exposure for 30 min elicited a marked response characterised by a decrease in compounds with antioxidant activity and changes to saturated fatty acid abundance – potentially to increase membrane stability under oxidative stress (Hu et al., 2018; Rawat et al., 2021). Such an outcome aligns with previous observations of metabolic reorganisation and acclimation over fast time scales, where coral photosynthesis is well known to adjust to high light stress exposure over minutes to hours (e.g., Gorbunov et al., 2001), as well as days (Downs et al., 2013; Lohr et al., 2019) and months (Cohen & Dubinsky, 2015). Metabolic changes under high light stress are not limited to Symbiodiniaceae photochemistry, yet fluorometric measurements of photochemical efficiency are often used as the only measure of coral health. Rapid metabolic reorganisation that occurs under stress is associated with high energetic costs (Kochman et al., 2021) and has impacts on long term health; here we demonstrate these changes may be better detected through utilisation of a paired metabolomic-volatilomic approach.

Changes in several compounds with known antioxidant properties indicated potential transient oxidative stress in our experiment. When corals are subjected to high light, excess light energy can inhibit carbon-fixation mechanisms and increase generation of ROS (Szabó et al., 2020). Excess energy can be dissipated via alternative electron pathways to prevent photodamage, though this comes at a cost of increased production of ROS (such as superoxide anions produced via the PSI Mehler reaction; Roberty et al., 2014) – driving oxidative stress that may ultimately trigger coral bleaching (Lesser, 2011). Among their many roles, sugar alcohols, such as sorbitol, inositol, and galactitol, are believed to facilitate ~ 30% of gross primary production instead of carbohydrates in terrestrial plants (Bieleski, 1982; Dumschott et al., 2017) and to behave as antioxidants in higher plants (Bhattacharya & Kundu, 2020; Nishizawa-Yokoi et al., 2008; Williamson et al., 2002). The marked decrease in abundance of two sugar alcohols, sorbitol and galactitol, suggested oxidative stress and a subsequent antioxidant response in our study. Additionally, nonanal (an aldehyde and fatty acid derivative; Tavassoli-Kafrani et al., 2016) and 2,5-cyclohexadiene-1,4-dione, 2,6-bis(1,1-dimethylethyl)- (a ketone), also decreased under high light, and may similarly be involved in antioxidant activity; aldehydes and ketones are both known to increase under oxidative stress in higher plants (Havaux, 2013). Notably, while we found a decrease in the abundance of compounds involved in maintaining the oxidative state of Symbiodiniaceae, an increase in sugar alcohols has been observed in host tissues under heat stress (Hillyer et al., 2017; Sogin et al., 2016), together with a higher abundance of nonanal released from the holobiont under thermal stress (Lawson et al., 2020). The onset of oxidative stress can be far more rapid when corals are subjected to light stress versus heat stress (Downs et al., 2013). The number of functional PSII reaction centres decrease rapidly, often resulting in compensatory increases in electron turnover rates through remaining functional centres to maintain overall photosynthetic performance (Behrenfeld et al., 1998; Gorbunov et al., 2001; Hennige et al., 2009). Hence, the observed metabolic response to light stress may reflect a more acute onset of oxidative stress than that observed in temperature stress studies.

We observed an increase in the abundance of two fatty acids (saturated myristic acid and mono-unsaturated oleic acid) under high light stress, while nonanal and oleamide (fatty acid derivatives) decreased. This increase in fatty acids in conjunction with a decrease in fatty acid derivatives suggests: (1) less cleavage of fatty acids and reduced formation of fatty acid derivatives (Dudareva et al., 2013; von Xylander et al., 2023); (2) an increased saturation of membrane-bound fatty acids to maintain membrane stability under oxidative stress (Safuan et al., 2021); or (3) the rate of degradation of fatty acid derivatives surpassed their rate of production. Previous studies under heat (Hillyer et al., 2017) and light (Lohr et al., 2019) stress suggest that increases in fatty acid abundance are likely the result of increased turnover of lipid stores, where translocation of key photosynthates such as carbohydrates are diminished under stress (Hillyer et al., 2017; Lohr et al., 2019). In contrast, whilst we cannot infer changes in translocation between partners from our current study, as our approach did not analyse host and symbiont separately, the observed accumulation of carbohydrates (> 5-fold increase in sugars such as tagatose, sorbose, fructose and arabinose) alongside an increase in fatty acids may indicate that the light stress was not long enough to elicit a full switch between carbohydrate and lipid driven energy modes (Hillyer et al., 2017; Lohr et al., 2019). However, this potential lack of transition to alternate energy sources does not equate to a lack of stress response elicited via transient high light stress and warrants further investigation. Furthermore, the transient light regime shifts imposed here (30 min) are shorter (albeit more intense, with treatment light levels being approximately eight times higher than that of the control) than for previous studies. For example, Lohr et al. (2019) took measurements after 7 days and 21 days of light shifts (with their “high-light” corals held at light levels ~ 4× that of “low-light” corals), and therefore, may capture different metabolic alterations that contrast with some of our findings. Specifically, Lohr et al. (2019) observed a decrease in glycerol abundance under high light, while an increase was observed in our study. This further reinforces that the light exposure used in the present study was indeed enough to elicit a transient stress or shock response (evidenced by decreased photochemical efficiency and changes to compounds involved in oxidative stress) but was not extensive enough to result in a complete change of energy strategy or photophysiological dysfunction. However, we note that the light levels used here may ultimately result in mortality before a change in energetic modes is observed, should the experiment duration be extended. While we observed a decrease in the abundance of sugar alcohols under transient light stress, Lohr et al. (2019) similarly noted a decrease in abundance of other sugar alcohols after 21 days under high light, but an increase in sugar alcohols after only 7 days. Such contrasting observations suggest an immediate oxidative stress response in which metabolites with antioxidant capacity are exhausted faster than they can be produced. Thus, how high light stress drives such changes should be examined over longer time scales (and under different intensities) with greater frequency of measurements to understand how metabolic processes are continuously fine-tuned over extended periods of sub-optimality and as organisms ultimately acclimate to their new growth conditions.

The correlative approach applied here tentatively linked volatile compounds to metabolic pathways for the first time. When applied to only metabolites, our correlative approach revealed that 45% of compounds that significantly correlated to each other belonged to the same metabolic pathways. We therefore used the same correlative approach, but this time combined the metabolite and volatile datasets to tentatively assign uncharacterised volatiles to metabolic pathways. Galactitol and sorbitol were correlated to 12 volatile compounds (benzene, (2,4-cyclopentadien-1-ylidenemethyl)-, benzyl alcohol, 2,5-cyclohexadiene-1,4-dione, 2,6-bis(1,1-dimethylethyl)-, cyclooctane-methyl, nonanal, bromodichloromethane, sulfurous acid, 2-ethylhexyl isohexyl ester and three unknown compounds) – as well as each other – and this cluster of compounds decreased under high light. In this case, we can tentatively propose that these compounds may have origins in galactose metabolism or related pathways, where sorbitol and galactitol are products of galactose breakdown (Zhang et al., 2020). Additionally, given the known antioxidant nature of sugar alcohols (such as galactitol and sorbitol; Keunen et al., 2013) and nonanal (Khan et al., 2018), it is also possible that the compounds within this correlation network may play a role in combating oxidative stress. The correlative approach used here could help elucidate the relationship between these metabolite and volatile compounds; similar correlative approaches have even been used to identify microbial interactions with metabolic pathways (Ni et al., 2020). However, such correlations may require validation by stable isotope tracking or biochemical approaches. We propose that, should this method be adopted more broadly in future and recurring correlations are identified, this approach could assist in identifying candidate compounds for further validation. This may ultimately allow us to determine the identity, origin, and function of many elusive, but potentially important, volatile compounds in corals. In turn, better knowledge of volatiles may equate to enhanced understanding of the metabolic processes that underlie the coral stress response, bleaching and mortality.

Our multi-omics approach has revealed how transient light stress elicits a significant metabolic response in *Montipora mollis*, and demonstrates how such an approach may better reveal changes in metabolism than traditional metabolomics alone. This response is characterised by an increase in carbohydrate and saturated fatty acid abundance, a decrease in abundance of compounds with antioxidant properties (possibly through utilisation in an oxidative stress response) and shares many biochemical similarities to those observed in corals under heat stress and longer-term light stress. Our results support previous studies highlighting that light stress may induce oxidative stress faster than heat stress, resulting in a more rapid antioxidant response, and while even transient light stress can trigger oxidative stress, longer term light stress may be required for a breakdown in translocation of photosynthates and an increase in catabolism of lipid stores as an alternate energy source. Through the integration of metabolomics and volatilomics datasets, seven volatiles were tentatively assigned to galactose metabolism pathways due to their correlation to sorbitol and galactitol, and we propose that our integrated approach can help elucidate the roles that undescribed volatile compounds play in coral metabolism. Doing so could enhance understanding of the molecular mechanisms underpinning coral stress responses (including bleaching). These findings provide direction for future work, including methodology for the integration of metabolomic and volatilomic analyses to help describe physiological and metabolic responses under different types of stressors.

### Electronic supplementary material

Below is the link to the electronic supplementary material.

Supplementary Material 1

Supplementary Material 2

## Data Availability

All raw metabolomics and volatilomics data files are available on the MassIVE mass spectrometry data repository (MSV000094920; 10.25345/C5J960N1V).
