# Supplementary figures and images for: Paired metabolomics and volatilomics provides insight into transient high light stress response mechanisms of the coral Montipora mollis
Source: Metabolomics. 2024 Jun 17;20(4):66. doi: 10.1007/s11306-024-02136-9 (PMC11182861; doi:10.1007/s11306-024-02136-9)

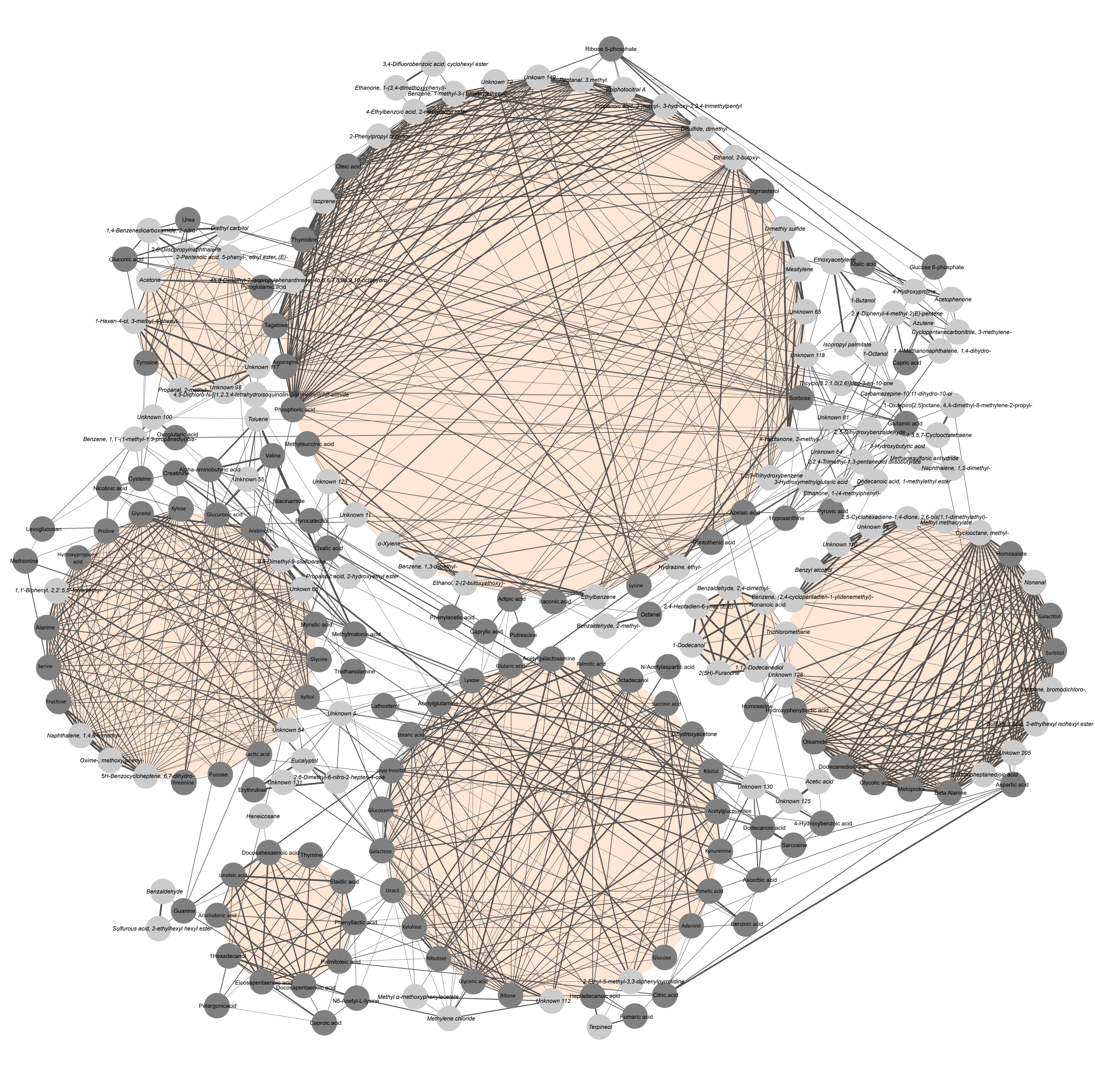

Supplement: Supplementary file 1 — Supplementary Material 1 [file 11306_2024_2136_MOESM1_ESM.pdf]
