# Supplementary material for: Paired metabolomics and volatilomics provides insight into transient high light stress response mechanisms of the coral Montipora mollis
Source: Metabolomics. 2024 Jun 17;20(4):66. doi: 10.1007/s11306-024-02136-9 (PMC11182861; doi:10.1007/s11306-024-02136-9)
Supplement: Supplementary file 2 — Supplementary Material 2 [file 11306_2024_2136_MOESM2_ESM.docx]

**Supplementary Information**

**for**

Paired metabolomics and volatilomics provides insight into transient light stress response mechanisms of the coral *Montipora mollis*

Natasha Bartels^1^*, Jennifer L. Matthews^1^, Caitlin A. Lawson^2^, Malcolm Possell^3^, David J. Hughes^4^, Jean-Baptiste Raina^1^, David J. Suggett^5^

^1^ Climate Change Cluster, Faculty of Science, University of Technology Sydney, Ultimo, New South Wales, Australia

^2^ Heron Island Research Station, Faculty of Science, University of Queensland, Gladstone, 4680, Australia

^3^ School of Life and Environmental Sciences, University of Sydney, Sydney, New South Wales, Australia

^4^Australian Institute of Marine Science, Townsville, Queensland, Australia

^5^ KAUST Reefscape Restoration Initiative (KRRI) and Red Sea Research Center (RSRC), King Abdullah University of Science and Technology, Thuwal, Saudi Arabia

*Correspondence: natasha.s.bartels@student.uts.edu.au

**Figure S1. Principal coordinate analysis and PERMANOVA of the volatilome and metabolome of control versus high-light corals.** The first and second principal coordinates account for (A) 52.25% of the total variance in the volatilome and (B) 51.02% in the metabolome.

**
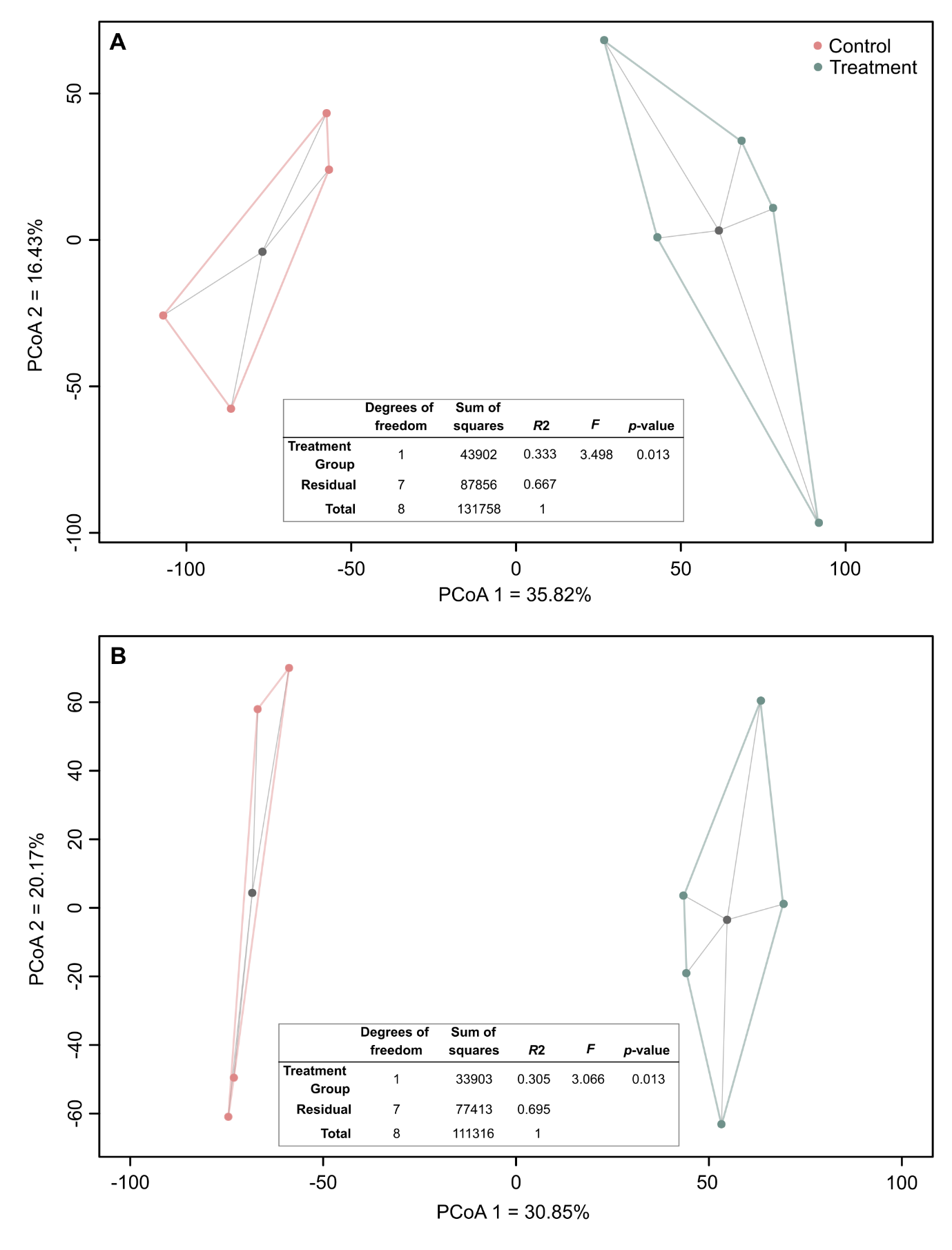
**

**Table S1. Validation steps taken to determine if correlations between volatile metabolites to metabolites could be used to tentatively map volatile metabolites to metabolic pathways.** For each metabolite, the number of correlations to other metabolites was calculated (“Total correlations”). For each of these correlations, it was determined whether these metabolites also appeared on the same KEGG pathway. Finally, the number of correlations with shared KEGG pathways was as a percentage of the total correlations was calculated. The mean of these values was ~45%.

| Compound | KEGG identifier | Total correlations | Correlations with shared KEGG pathway | Correlations with shared KEGG pathways (%) |
| --- | --- | --- | --- | --- |
| Phosphoric acid | C00009 | 11 | 3 | 27.27 |
| Pyruvic acid | C00022 | 4 | 4 | 100.00 |
| Glutamic acid | C00025 | 4 | 3 | 75.00 |
| Oxoglutaric acid | C00026 | 6 | 2 | 33.33 |
| Glycine | C00037 | 13 | 10 | 76.92 |
| Alanine | C00041 | 14 | 11 | 78.57 |
| Succinic acid | C00042 | 11 | 10 | 90.91 |
| Lysine | C00047 | 6 | 4 | 66.67 |
| Aspartic acid | C00049 | 5 | 4 | 80.00 |
| Glutamine | C00064 | 0 | 0 | - |
| Serine | C00065 | 14 | 10 | 71.43 |
| Methionine | C00073 | 6 | 3 | 50.00 |
| Ornithine | C00077 | 0 | 0 | - |
| Tryptophan | C00078 | 0 | 0 | - |
| Tyrosine | C00082 | 4 | 3 | 75.00 |
| Urea | C00086 | 2 | 0 | 0.00 |
| Sucrose | C00089 | 0 | 0 | - |
| Glucose 6-phosphate | C00092 | 3 | 2 | 66.67 |
| Glycerol 3-phosphate | C00093 | 0 | 0 | - |
| Cysteine | C00097 | 6 | 4 | 66.67 |
| Beta-Alanine | C00099 | 9 | 6 | 66.67 |
| Uracil | C00106 | 16 | 12 | 75.00 |
| Glycerol | C00116 | 9 | 5 | 55.56 |
| Ribose 5-phosphate | C00117 | 5 | 3 | 60.00 |
| Ribose | C00121 | 22 | 17 | 77.27 |
| Fumaric acid | C00122 | 2 | 1 | 50.00 |
| Putrescine | C00134 | 8 | 5 | 62.50 |
| myo-Inositol | C00137 | 12 | 9 | 75.00 |
| Acetyl D-glucosamine | C00140 | 8 | 4 | 50.00 |
| Adenine | C00147 | 9 | 5 | 55.56 |
| Proline | C00148 | 14 | 6 | 42.86 |
| Asparagine | C00152 | 11 | 6 | 54.55 |
| Niacinamide | C00153 | 3 | 2 | 66.67 |
| 4-Hydroxybenzoic acid | C00156 | 4 | 2 | 50.00 |
| Citric acid | C00158 | 5 | 2 | 40.00 |
| Thymine | C00178 | 9 | 3 | 33.33 |
| Xylose | C00181 | 14 | 8 | 57.14 |
| Valine | C00183 | 11 | 7 | 63.64 |
| Dihydroxyacetone | C00184 | 10 | 6 | 60.00 |
| Lactic acid | C00186 | 9 | 3 | 33.33 |
| Threonine | C00188 | 11 | 7 | 63.64 |
| Glucuronic acid | C00191 | 13 | 6 | 46.15 |
| Maltose | C00208 | 0 | 0 | - |
| Oxalic acid | C00209 | 5 | 3 | 60.00 |
| Adenosine | C00212 | 0 | 0 | - |
| Sarcosine | C00213 | 4 | 1 | 25.00 |
| Thymidine | C00214 | 10 | 4 | 40.00 |
| Arabinose | C00216 | 9 | 4 | 44.44 |
| Arachidonic acid | C00219 | 12 | 6 | 50.00 |
| Glucose | C00221 | 7 | 3 | 42.86 |
| Guanine | C00242 | 5 | 0 | 0.00 |
| Alpha Lactose | C00243 | 0 | 0 | - |
| Palmitic acid | C00249 | 9 | 7 | 77.78 |
| Nicotinic acid | C00253 | 6 | 1 | 16.67 |
| Gluconic acid | C00257 | 3 | 0 | 0.00 |
| Glyceric acid | C00258 | 18 | 8 | 44.44 |
| Hypoxanthine | C00262 | 5 | 3 | 60.00 |
| Homoserine | C00263 | 3 | 2 | 66.67 |
| Ribulose | C00309 | 12 | 4 | 33.33 |
| Xylulose | C00310 | 12 | 3 | 25.00 |
| Kynurenine | C00328 | 9 | 5 | 55.56 |
| Glucosamine | C00329 | 13 | 3 | 23.08 |
| Xylitol | C00379 | 7 | 1 | 14.29 |
| Xanthine | C00385 | 0 | 0 | - |
| Mannitol | C00392 | 0 | 0 | - |
| Tryptamine | C00398 | 0 | 0 | - |
| Isoleucine | C00407 | 0 | 0 | - |
| Ribitol | C00474 | 11 | 4 | 36.36 |
| Tyramine | C00483 | 0 | 0 | - |
| Glutaric acid | C00489 | 10 | 1 | 10.00 |
| Itaconic acid | C00490 | 3 | 1 | 33.33 |
| Acetylmannosamine | C00645 | 0 | 0 | - |
| Oleic acid | C00712 | 12 | 1 | 8.33 |
| Creatinine | C00791 | 4 | 0 | 0.00 |
| Sorbitol | C00794 | 9 | 3 | 33.33 |
| Tagatose | C00795 | 9 | 2 | 22.22 |
| 1-Hexadecanol | C00823 | 10 | 0 | 0.00 |
| Pantothenic acid | C00864 | 4 | 0 | 0.00 |
| Indoleacetic acid | C00954 | 0 | 0 | - |
| Galactose | C00984 | 13 | 1 | 7.69 |
| Hydroxypropionic acid | C01013 | 13 | 1 | 7.69 |
| Ascorbic acid | C01041 | 5 | 2 | 40.00 |
| N-Acetyl L-aspartic acid | C01042 | 3 | 0 | 0.00 |
| Trehalose | C01083 | 0 | 0 | - |
| 3-Hydroxybutyric acid | C01089 | 5 | 1 | 20.00 |
| 1,2,3-Trihydroxybenzene | C01108 | 6 | 0 | 0.00 |
| 4-Hydroxyproline | C01157 | 4 | 1 | 25.00 |
| Lathosterol | C01189 | 4 | 2 | 50.00 |
| Phenyllactic acid | C01479 | 19 | 0 | 0.00 |
| Stearic acid | C01530 | 10 | 1 | 10.00 |
| Capric acid | C01571 | 10 | 0 | 0.00 |
| Linoleic acid | C01595 | 13 | 5 | 38.46 |
| Threonic acid | C01620 | 0 | 0 | - |
| Galactitol | C01697 | 9 | 1 | 11.11 |
| Pyroglutamic acid | C01879 | 4 | 0 | 0.00 |
| Methylmalonic acid | C02170 | 13 | 1 | 7.69 |
| Cystathionine | C02291 | 0 | 0 | - |
| Fructose | C02656 | 11 | 0 | 0.00 |
| Dodecanoic acid | C02679 | 7 | 0 | 0.00 |
| Acetyllysine | C02727 | 10 | 1 | 10.00 |
| Dopamine | C03758 | 0 | 0 | - |
| Stigmasterol | C05442 | 11 | 0 | 0.00 |
| Adipic acid | C06104 | 9 | 1 | 11.11 |
| Caprylic acid | C06423 | 8 | 0 | 0.00 |
| Myristic acid | C06424 | 9 | 0 | 0.00 |
| Eicosapentaenoic acid | C06428 | 14 | 2 | 14.29 |
| Docosahexaenoic acid | C06429 | 9 | 1 | 11.11 |
| Phenylacetic acid | C07086 | 1 | 0 | 0.00 |

**Table S2. Tukey’s HSD performed on photochemical efficiency data, comparing control and treatment before and directly after the transient high light event, and after 30 minutes recovery.** Significant values are bold.

| Comparison | Difference in Means | Lower | Upper | p-value |
| --- | --- | --- | --- | --- |
| Before high light – Treatment vs Control | 0.041 | -0.088 | 0.170 | 0.888 |
| Directly after high light - Treatment vs Control | -0.190 | -0.319 | -0.061 | 0.003 |
| After 30 min recovery - Treatment vs Control | -0.074 | -0.203 | 0.054 | 0.425 |

**Table S3. Principal component loadings of volatiles driving differences between control and treatment groups.**

| Compound | PC1 (44.3%) | PC2 (17.4%) |
| --- | --- | --- |
| Benzene, 1,1'-(1-methyl-1,3-propanediyl)bis- | 0.016 | -0.283 |
| Terpineol | 0.072 | -0.201 |
| 2-Ethyl-5-methyl-3,3-diphenylpyrrolidine | 0.024 | -0.193 |
| Unknown 54 | 0.053 | -0.191 |
| 1,1'-Biphenyl, 2,2',5,5'-tetramethyl- | -0.096 | -0.172 |
| 2-Pentenoic acid, 5-phenyl-, ethyl ester, (E)- | 0.037 | -0.158 |
| 1,3-Dioxan-5-ol, 2-phenyl- | -0.018 | -0.142 |
| Naphthalene, 1,4,6-trimethyl- | 0.149 | -0.137 |
| Phenol | -0.044 | -0.133 |
| Unknown 55 | 0.019 | -0.133 |
| Eucalyptol | 0.190 | -0.116 |
| Unknown 117 | 0.189 | -0.116 |
| Benzene, (2,4-cyclopentadien-1-ylidenemethyl)- | -0.165 | -0.105 |
| Unknown 110 | -0.139 | -0.090 |
| Unknown 98 | 0.236 | -0.087 |
| 2-Phenylpropyl butyrate | 0.144 | -0.084 |
| Benzyl alcohol | -0.134 | -0.081 |
| Unknown 83 | -0.123 | -0.074 |
| Homosalate | -0.137 | -0.060 |
| Nonanal | -0.174 | -0.055 |
| Methane, bromodichloro- | -0.123 | -0.026 |
| Unknown 72 | 0.192 | -0.021 |
| Unknown 81 | 0.167 | -0.015 |
| 2,2,4-Trimethyl-1,3-pentanediol diisobutyrate | -0.190 | -0.008 |
| Unknown 118 | 0.123 | -0.001 |
| Methyl Œ±-methoxyphenylacetate | 0.118 | 0.011 |
| Cyclopentanecarbonitrile, 3-methylene- | 0.120 | 0.018 |
| 4-Heptanone, 2-methyl- | 0.047 | 0.119 |
| Acetone | 0.086 | 0.121 |
| 1-Butanol | 0.090 | 0.121 |
| Heneicosane | 0.033 | 0.154 |
| o-Xylene | -0.155 | 0.168 |
| Azulene | -0.201 | 0.179 |
| Dimethly sulfide | 0.040 | 0.180 |
| Isopropyl palmitate | 0.080 | 0.184 |
| Ethoxyacetylene | 0.108 | 0.196 |
| Hydrazine, ethyl- | 0.048 | 0.209 |

**Table S4. Principal components loadings of metabolites driving differences between control and treatment groups.**

| Compound | PC1 (80.9%) | PC2 (16.4%) |
| --- | --- | --- |
| Pyruvic acid | 0.011 | 0.000 |
| Lactic acid | 0.000 | -0.059 |
| Glycolic acid | -0.183 | 0.054 |
| Caproic acid | 0.020 | 0.027 |
| Alanine | -0.006 | -0.139 |
| Glycine | 0.009 | -0.076 |
| Oxalic acid | 0.011 | 0.026 |
| Sarcosine | 0.013 | -0.019 |
| Hydroxypropionic acid | 0.018 | -0.073 |
| 3-Hydroxybutyric acid | 0.024 | 0.042 |
| Alpha-aminobutyric acid | -0.008 | -0.211 |
| Beta-Alanine | -0.161 | 0.037 |
| Methylmalonic acid | -0.007 | -0.101 |
| Valine | -0.006 | -0.104 |
| Urea | 0.015 | -0.041 |
| Dihydroxyacetone | 0.016 | -0.015 |
| Benzoic acid | 0.009 | -0.031 |
| Caprylic acid | 0.008 | 0.251 |
| Glycerol | 0.159 | -0.089 |
| Phosphoric acid | 0.183 | 0.124 |
| Nicotinic acid | -0.005 | -0.067 |
| Proline | -0.003 | -0.166 |
| Phenylacetic acid | 0.011 | 0.032 |
| Succinic acid | -0.002 | 0.023 |
| Methylsuccinic acid | -0.003 | 0.009 |
| Glyceric acid | -0.009 | -0.018 |
| Pyrocatechol | 0.014 | 0.161 |
| Fumaric acid | 0.014 | 0.048 |
| Uracil | -0.020 | 0.034 |
| Itaconic acid | -0.007 | 0.003 |
| Pelargonic acid | 0.003 | 0.157 |
| Serine | -0.002 | -0.150 |
| Threonine | -0.001 | -0.182 |
| Glutaric acid | 0.020 | -0.045 |
| Thymine | -0.017 | 0.141 |
| Homoserine | -0.222 | 0.066 |
| Capric acid | 0.006 | 0.039 |
| Erythrulose | 0.171 | -0.127 |
| Malic acid | 0.005 | -0.036 |
| Niacinamide | 0.038 | -0.110 |
| Adipic acid | 0.005 | 0.075 |
| Aspartic acid | -0.148 | 0.038 |
| Methionine | -0.032 | -0.178 |
| 4-Hydroxyproline | 0.177 | -0.083 |
| Pyroglutamic acid | -0.005 | -0.052 |
| 1,2,3-Trihydroxybenzene | 0.192 | 0.097 |
| Cysteine | 0.011 | -0.145 |
| Creatinine | -0.003 | -0.096 |
| Oxoglutaric acid | -0.014 | -0.262 |
| Pimelic acid | 0.016 | 0.036 |
| Phenyllactic acid | -0.009 | 0.089 |
| 3-Hydroxymethylglutaric acid | -0.007 | 0.101 |
| Triethanolamine | -0.026 | -0.026 |
| Glutamic acid | -0.015 | 0.012 |
| 4-Hydroxybenzoic acid | 0.014 | 0.053 |
| Dodecanoic acid | 0.006 | -0.009 |
| N-Acetyl-L-aspartic acid | 0.033 | 0.026 |
| Lyxose | -0.034 | -0.058 |
| Xylose | 0.021 | -0.161 |
| Arabinose | 0.145 | -0.070 |
| Asparagine | 0.162 | 0.024 |
| Ribulose | -0.007 | 0.076 |
| Ribose | 0.004 | -0.030 |
| Xylulose | -0.004 | 0.060 |
| Xylitol | 0.023 | -0.114 |
| Levoglucosan | 0.015 | -0.051 |
| Ribitol | 0.005 | -0.075 |
| Fucose | 0.004 | -0.045 |
| Putrescine | -0.009 | 0.170 |
| Azelaic acid | -0.015 | 0.171 |
| 2-Aminoheptanedioic acid | -0.154 | 0.015 |
| Hypoxanthine | 0.000 | 0.090 |
| Citric acid | -0.158 | 0.017 |
| Myristic acid | 0.164 | -0.072 |
| Tagatose | 0.186 | -0.089 |
| Fructose | 0.158 | -0.129 |
| Sorbose | 0.184 | -0.065 |
| Adenine | 0.004 | 0.014 |
| Hydroxyphenyllactic acid | -0.169 | 0.055 |
| Glucose | 0.008 | -0.004 |
| N-Acetylglutamine | 0.006 | -0.003 |
| Lysine | 0.179 | 0.102 |
| Glucosamine | 0.000 | -0.033 |
| Galactose | 0.008 | -0.061 |
| N6-Acetyl-L-lysine | 0.003 | 0.075 |
| 1-Hexadecanol | 0.007 | 0.099 |
| Tyrosine | 0.045 | -0.080 |
| Glucuronic acid | 0.015 | -0.117 |
| Sorbitol | -0.168 | 0.009 |
| Galactitol | -0.156 | -0.005 |
| Ascorbic acid | -0.001 | -0.006 |
| Galacturonic acid | -0.151 | -0.004 |
| Pantothenic acid | 0.008 | 0.012 |
| Palmitoleic acid | 0.011 | 0.068 |
| Gluconic acid | -0.007 | -0.014 |
| Palmitic acid | 0.010 | 0.027 |
| Dodecanedioic acid | -0.156 | 0.034 |
| N-Acetyl-D-glucosamine | -0.003 | -0.025 |
| Ribose 5-phosphate | 0.201 | 0.063 |
| myo-Inositol | 0.000 | -0.032 |
| N-Acetylgalactosamine | -0.001 | -0.024 |
| Heptadecanoic acid | 0.015 | 0.102 |
| Guanine | 0.189 | 0.109 |
| Kynurenine | 0.009 | -0.028 |
| Octadecanol | 0.013 | 0.040 |
| Linoleic acid | 0.034 | 0.109 |
| Oleic acid | 0.166 | 0.071 |
| Elaidic acid | 0.003 | 0.121 |
| Stearic acid | 0.009 | 0.081 |
| Metoprolol | -0.199 | 0.058 |
| Glucose 6-phosphate | 0.231 | 0.111 |
| Arachidonic acid | -0.020 | 0.087 |
| Eicosapentaenoic acid | -0.008 | 0.121 |
| Oleamide | -0.148 | 0.028 |
| Thymidine | 0.176 | 0.078 |
| Stigmasterol | 0.262 | 0.190 |
| Docosahexaenoic acid | -0.018 | 0.110 |
| Docosapentaenoic acid | -0.021 | 0.170 |
| Lathosterol | 0.010 | -0.033 |

**Table S5. Relative abundance and fold-change of all identified volatiles compounds in control versus treatment (high light) groups.** Where data passed all quality control and filtering steps, the fold change between control and treatment and FDR corrected p-values are included. Significant values are in bold.

| **Compound** | **Control** | | | | **Treatment** | | | | | **Fold change** | **FDR corrected p-value** |
| --- | --- | --- | --- | --- | --- | --- | --- | --- | --- | --- | --- |
| 1,5-Methano-8H-pyrido[1,2-a][1,5]diazocin-8-one, decahydro-4-(2-propenyl)-, [1S-(1α,4α,5α,11aα)]- | 0.000 | 0.000 | 0.000 | 0.000 | 0.000 | 0.000 | 0.016 | 0.000 | 0.000 | - | - |
| 11-Oxo-5,5-dimethyl-5-sila-11H-5,10-dihydrobenzo[b]pyrido[4,3-e]azepine | 0.001 | 0.000 | 0.000 | 0.000 | 0.000 | 0.000 | 0.000 | 0.000 | 0.000 | - | - |
| Propanoic acid, 2-methyl-, 3-hydroxy-2,2,4-trimethylpentyl | 0.038 | 0.000 | 0.000 | 0.000 | 0.009 | 0.042 | 0.048 | 0.032 | 0.000 | 0.103 | 0.064 |
| [1,1'-Bicyclopentyl]-2-one | 0.000 | 0.000 | 0.007 | 0.005 | 0.000 | 0.000 | 0.000 | 0.000 | 0.002 | - | - |
| 1,12-Dodecanediol | 0.004 | 0.000 | 0.005 | 0.004 | 0.000 | 0.001 | 0.000 | 0.001 | 0.000 | 5.554 | 0.059 |
| 1,13-Tetradecadiene | 0.004 | 0.000 | 0.004 | 0.000 | 0.000 | 0.000 | 0.000 | 0.000 | 0.000 | - | - |
| 1,1'-Biphenyl, 2,2',5,5'-tetramethyl- | 0.001 | 0.001 | 0.003 | 0.017 | 0.000 | 0.000 | 0.000 | 0.000 | 0.002 | 16.383 | 0.134 |
| 1,2,3-Trihydroxybenzene | 0.004 | 0.003 | 0.000 | 0.003 | 0.004 | 0.003 | 0.002 | 0.001 | 0.004 | 0.097 | **0.000** |
| 1,3,5,7-Cyclooctatetraene | 0.000 | 0.020 | 0.001 | 0.001 | 0.000 | 0.000 | 0.000 | 0.035 | 0.000 | 15.070 | 0.102 |
| 1,3,5-Trifluorobenzene | 0.000 | 0.000 | 0.013 | 0.000 | 0.000 | 0.000 | 0.000 | 0.000 | 0.000 | - | - |
| 1,3-Dioxan-5-ol, 2-phenyl- | 0.000 | 0.003 | 0.003 | 0.006 | 0.004 | 0.000 | 0.002 | 0.000 | 0.003 | 2.102 | 0.717 |
| 1,4-Benzenedicarboxamide, 2-nitro- | 0.000 | 0.005 | 0.006 | 0.005 | 0.029 | 0.006 | 0.029 | 0.000 | 0.008 | 0.327 | 0.678 |
| 1,4-Methanonaphthalene, 1,4-dihydro- | 0.002 | 0.006 | 0.001 | 0.004 | 0.004 | 0.002 | 0.003 | 0.002 | 0.002 | 1.374 | 0.522 |
| 1-Butanol | 0.005 | 0.000 | 0.015 | 0.000 | 0.000 | 0.006 | 0.001 | 0.009 | 0.017 | 0.051 | 0.069 |
| 1-Dodecanol, TMS derivative | 0.001 | 0.000 | 0.005 | 0.005 | 0.000 | 0.000 | 0.001 | 0.000 | 0.000 | 28.018 | 0.083 |
| 1-Hexadecanol | 3.074 | 1.898 | 1.496 | 1.723 | 2.213 | 2.408 | 1.074 | 0.681 | 3.085 | 0.927 | 0.821 |
| 1-Hexanol, 2-ethyl- | 0.002 | 0.000 | 0.236 | 0.000 | 0.000 | 0.000 | 0.009 | 0.000 | 0.000 | - | - |
| 1-Hexen-4-ol, 3-methyl-4-phenyl- | 0.000 | 0.000 | 0.008 | 0.104 | 0.000 | 0.046 | 0.006 | 0.000 | 0.004 | 0.049 | 0.245 |
| 1-Hexene, 3-methyl- | 0.000 | 0.000 | 0.000 | 0.001 | 0.000 | 0.000 | 0.000 | 0.000 | 0.005 | - | - |
| 1-Methyl-4-isopropyl-cyclohexyl 2-hydroperfluorobutanoate | 0.000 | 0.000 | 0.001 | 0.000 | 0.003 | 0.000 | 0.000 | 0.000 | 0.000 | - | - |
| 1-Octanol | 0.007 | 0.008 | 0.005 | 0.005 | 0.002 | 0.003 | 0.006 | 0.005 | 0.001 | 2.159 | 0.122 |
| 1-Octanol, 2-butyl- | 0.000 | 0.000 | 0.000 | 0.000 | 0.028 | 0.000 | 0.000 | 0.000 | 0.000 | - | - |
| 1-Oxaspiro[2.5]octane, 4,4-dimethyl-8-methylene-2-propyl- | 0.000 | 0.002 | 0.001 | 0.001 | 0.000 | 0.001 | 0.000 | 0.000 | 0.000 | 5.394 | 0.059 |
| 1-Propene, 2-methyl- | 0.000 | 0.000 | 0.005 | 0.000 | 0.000 | 0.000 | 0.000 | 0.000 | 0.003 | - | - |
| 2-(2-Methoxyethoxy)ethyl acetate | 0.000 | 0.000 | 0.003 | 0.002 | 0.000 | 0.000 | 0.000 | 0.000 | 0.000 | - | - |
| 2(5H)-Furanone | 0.004 | 0.000 | 0.007 | 0.006 | 0.000 | 0.000 | 0.000 | 0.000 | 0.000 | 7.060 | 0.061 |
| 2,2,4-Trimethyl-1,3-pentanediol diisobutyrate | 0.063 | 0.098 | 0.078 | 0.086 | 0.000 | 0.022 | 0.004 | 0.002 | 0.002 | 15.662 | **0.020** |
| 2,2-Diethoxyacetophenone | 0.000 | 0.019 | 0.006 | 0.000 | 0.000 | 0.004 | 0.000 | 0.000 | 0.005 | - | - |
| 2,4,6-Trimethoxystyrene | 0.000 | 0.000 | 0.006 | 0.000 | 0.000 | 0.000 | 0.000 | 0.000 | 0.000 | - | - |
| 2,4-Diphenyl-4-methyl-2(E)-pentene | 0.013 | 0.063 | 0.011 | 0.027 | 0.052 | 0.017 | 0.020 | 0.011 | 0.011 | 1.087 | 0.678 |
| 2,4-Heptadien-6-ynal, (E,E)- | 0.006 | 0.000 | 0.006 | 0.009 | 0.000 | 0.000 | 0.000 | 0.000 | 0.000 | 6.124 | 0.059 |
| 2,5-Cyclohexadiene-1,4-dione, 2,6-bis(1,1-dimethylethyl)- | 0.006 | 0.009 | 0.015 | 0.019 | 0.000 | 0.000 | 0.000 | 0.000 | 0.000 | 14.678 | **0.002** |
| 2,5-Dihydroxybenzaldehyde, 2TMS derivative | 0.006 | 0.008 | 0.007 | 0.015 | 0.000 | 0.009 | 0.020 | 0.015 | 0.008 | 1.322 | 0.687 |
| 2,6-Diisopropylnaphthalene | 0.000 | 0.094 | 0.036 | 0.029 | 0.074 | 0.038 | 0.038 | 0.000 | 0.014 | 1.014 | 0.966 |
| 2,6-Dimethyl-6-nitro-2-hepten-4-one | 0.000 | 0.000 | 0.022 | 0.000 | 0.042 | 0.025 | 0.026 | 0.000 | 0.000 | 0.199 | 0.189 |
| 2-Amino-1,3-propanediol | 0.000 | 0.000 | 0.019 | 0.050 | 0.000 | 0.015 | 0.000 | 0.000 | 0.033 | - | - |
| 2-Aminoheptanedioic acid | 0.058 | 0.041 | 0.059 | 0.038 | 0.053 | 0.028 | 0.046 | 0.038 | 0.038 | 6.591 | **0.000** |
| 2-Butanol | 0.000 | 0.000 | 0.000 | 0.000 | 0.000 | 0.000 | 0.000 | 0.000 | 0.013 | - | - |
| 2-Ethyl-5-methyl-3,3-diphenylpyrrolidine | 0.000 | 0.006 | 0.006 | 0.015 | 0.028 | 0.009 | 0.000 | 0.000 | 0.012 | 0.751 | 0.983 |
| 2-Hexenal, 6-phenyl-, (E)- | 0.000 | 0.000 | 0.000 | 0.018 | 0.000 | 0.000 | 0.000 | 0.000 | 0.009 | - | - |
| 2-Pentenoic acid, 5-phenyl-, ethyl ester, (E)- | 0.002 | 0.000 | 0.001 | 0.003 | 0.009 | 0.001 | 0.002 | 0.000 | 0.005 | 0.556 | 0.923 |
| 2-Phenylpropenal | 0.000 | 0.005 | 0.001 | 0.000 | 0.000 | 0.000 | 0.000 | 0.000 | 0.000 | - | - |
| 2-Phenylpropyl butyrate | 0.004 | 0.007 | 0.000 | 0.000 | 0.015 | 0.007 | 0.000 | 0.002 | 0.009 | 0.033 | 0.079 |
| 2-Propanamine, 2-methyl- | 0.000 | 0.000 | 0.000 | 0.000 | 0.000 | 0.003 | 0.000 | 0.000 | 0.006 | - | - |
| 2-Propanone, 1-hydroxy- | 0.112 | 0.000 | 0.000 | 0.059 | 0.000 | 0.127 | 0.000 | 0.190 | 0.000 | - | - |
| 2-Propenal | 0.000 | 0.000 | 0.004 | 0.011 | 0.000 | 0.000 | 0.000 | 0.000 | 0.007 | - | - |
| 3(2H)-Benzofuranone, 7-methyl- | 0.000 | 0.000 | 0.006 | 0.005 | 0.000 | 0.000 | 0.000 | 0.000 | 0.000 | - | - |
| 3,4,4-Trimethylcyclohexene | 0.000 | 0.000 | 0.010 | 0.000 | 0.000 | 0.000 | 0.000 | 0.000 | 0.000 | - | - |
| 3,4-Difluorobenzoic acid, cyclohexyl ester | 0.000 | 0.000 | 0.002 | 0.006 | 0.006 | 0.000 | 0.005 | 0.000 | 0.006 | 0.219 | 0.189 |
| 3-Hydroxymethylglutaric acid | 0.010 | 0.009 | 0.004 | 0.015 | 0.014 | 0.009 | 0.002 | 0.003 | 0.011 | 1.101 | 0.892 |
| 4,4'-Diacetyl biphenyl | 0.000 | 0.000 | 0.002 | 0.001 | 0.001 | 0.000 | 0.000 | 0.000 | 0.000 | - | - |
| 4,5-Dichloro-N-[(1,2,3,4-tetrahydroisoquinolin-2-yl)methyl]phthalimide | 0.000 | 0.006 | 0.007 | 0.000 | 0.002 | 0.004 | 0.002 | 0.000 | 0.001 | 0.092 | 0.064 |
| 4b,8-Dimethyl-2-isopropylphenanthrene, 4b,5,6,7,8,8a,9,10-octahydro- | 0.000 | 0.000 | 0.002 | 0.000 | 0.000 | 0.001 | 0.001 | 0.000 | 0.001 | 0.244 | 0.181 |
| 4-Ethylbenzoic acid, 2-methylbutyl ester | 0.000 | 0.000 | 0.014 | 0.024 | 0.026 | 0.000 | 0.006 | 0.000 | 0.006 | 0.083 | 0.230 |
| 4-Ethylbenzoic acid, tridec-2-ynyl ester | 0.000 | 0.000 | 0.000 | 0.000 | 0.000 | 0.000 | 0.000 | 0.007 | 0.009 | - | - |
| 4-Heptanone, 2-methyl- | 0.129 | 0.000 | 0.000 | 0.000 | 0.000 | 0.054 | 0.080 | 0.056 | 0.000 | 0.162 | 0.189 |
| 5,9-Undecadien-2-one, 6,10-dimethyl-, (E)- | 0.017 | 0.000 | 0.015 | 0.000 | 0.000 | 0.000 | 0.000 | 0.000 | 0.000 | - | - |
| 5H-Benzocycloheptene, 6,7-dihydro- | 0.000 | 0.000 | 0.000 | 0.003 | 0.004 | 0.001 | 0.000 | 0.000 | 0.004 | 0.137 | 0.192 |
| 8-Phenylisoquinoline | 0.000 | 0.000 | 0.000 | 0.002 | 0.000 | 0.000 | 0.000 | 0.001 | 0.000 | - | - |
| 9,9-Dimethyl-9-silafluorene | 0.000 | 0.000 | 0.004 | 0.000 | 0.009 | 0.007 | 0.000 | 0.000 | 0.002 | 0.072 | 0.205 |
| Acetic acid | 0.062 | 0.035 | 0.000 | 0.000 | 0.029 | 0.048 | 0.092 | 0.072 | 0.093 | 0.129 | **0.000** |
| Acetone | 0.939 | 0.000 | 0.012 | 0.000 | 0.000 | 0.011 | 0.288 | 0.000 | 0.007 | 0.017 | 0.272 |
| Acetophenone | 0.034 | 0.119 | 0.022 | 0.024 | 0.039 | 0.020 | 0.064 | 0.041 | 0.018 | 1.312 | 0.522 |
| Azulene | 0.003 | 0.013 | 0.003 | 0.011 | 0.000 | 0.001 | 0.005 | 0.002 | 0.000 | 5.408 | 0.175 |
| Benzaldehyde | 0.000 | 0.020 | 0.000 | 0.029 | 0.014 | 0.000 | 0.004 | 0.017 | 0.013 | 0.084 | 0.064 |
| Benzaldehyde, 2,4-dimethyl- | 0.003 | 0.000 | 0.014 | 0.029 | 0.000 | 0.000 | 0.000 | 0.000 | 0.000 | 26.056 | 0.083 |
| Benzaldehyde, 2-methyl- | 0.004 | 0.011 | 0.004 | 0.000 | 0.000 | 0.000 | 0.005 | 0.003 | 0.000 | 5.723 | 0.059 |
| Benzaldehyde, 4-methyl- | 0.003 | 0.011 | 0.000 | 0.000 | 0.000 | 0.000 | 0.004 | 0.000 | 0.000 | - | - |
| Benzene | 0.000 | 0.000 | 0.005 | 0.012 | 0.000 | 0.000 | 0.001 | 0.000 | 0.000 | - | - |
| Benzene, (2,4-cyclopentadien-1-ylidenemethyl)- | 0.005 | 0.002 | 0.009 | 0.016 | 0.000 | 0.000 | 0.000 | 0.000 | 0.000 | 51.758 | **0.004** |
| Benzene, 1,1'-(1,4-dimethyl-1-butene-1,4-diyl)bis- | 0.000 | 0.045 | 0.000 | 0.000 | 0.000 | 0.037 | 0.000 | 0.000 | 0.000 | - | - |
| Benzene, 1,1'-(1-methyl-1,3-propanediyl)bis- | 0.001 | 0.002 | 0.004 | 0.002 | 0.012 | 0.006 | 0.000 | 0.000 | 0.005 | 0.540 | 0.821 |
| benzene, 1,1'-(1-methylethylidene)bis[4-methyl- | 0.000 | 0.000 | 0.000 | 0.012 | 0.000 | 0.006 | 0.000 | 0.000 | 0.013 | - | - |
| Benzene, 1,2,3-trimethyl- | 0.000 | 0.000 | 0.000 | 0.000 | 0.000 | 0.000 | 0.000 | 0.000 | 0.002 | - | - |
| Benzene, 1,3-dimethyl- | 0.001 | 0.023 | 0.007 | 0.008 | 0.000 | 0.001 | 0.003 | 0.006 | 0.000 | 5.857 | 0.122 |
| Benzene, 1-methyl-3-(1-methylethenyl)- | 0.000 | 0.000 | 0.002 | 0.002 | 0.004 | 0.000 | 0.002 | 0.000 | 0.004 | 0.144 | 0.192 |
| Benzene, 2-methoxy-1-(2-nitroethenyl)-3-(phenylmethoxy)- | 0.011 | 0.032 | 0.000 | 0.000 | 0.000 | 0.013 | 0.000 | 0.012 | 0.000 | - | - |
| Benzothiazole | 0.000 | 0.000 | 0.012 | 0.029 | 0.000 | 0.003 | 0.000 | 0.000 | 0.009 | - | - |
| Benzyl alcohol | 0.004 | 0.003 | 0.006 | 0.011 | 0.000 | 0.000 | 0.000 | 0.000 | 0.000 | 21.517 | **0.002** |
| Benzyl sulfide | 0.000 | 0.000 | 0.000 | 0.013 | 0.009 | 0.000 | 0.000 | 0.000 | 0.000 | - | - |
| Bicyclo[3.2.0]hepta-2,6-diene | 0.000 | 0.000 | 0.004 | 0.006 | 0.000 | 0.000 | 0.006 | 0.002 | 0.000 | - | - |
| Bicyclo[4.1.0]heptane, 2-methyl- | 0.000 | 0.000 | 0.001 | 0.000 | 0.000 | 0.000 | 0.000 | 0.000 | 0.000 | - | - |
| Butane, 1-chloro-3-methyl- | 0.003 | 0.000 | 0.000 | 0.000 | 0.000 | 0.000 | 0.000 | 0.238 | 0.008 | - | - |
| Butanoic acid, 3-(1-phenylethoxy)- | 0.000 | 0.000 | 0.000 | 0.021 | 0.000 | 0.000 | 0.000 | 0.002 | 0.023 | - | - |
| Butyrolactone | 0.000 | 0.000 | 0.000 | 0.012 | 0.000 | 0.000 | 0.000 | 0.000 | 0.006 | - | - |
| Caprolactam | 0.000 | 0.000 | 0.000 | 0.000 | 0.000 | 0.000 | 0.000 | 0.000 | 0.010 | - | - |
| Carbamazepine-10,11-dihydro-10-ol,2TMS derivative | 0.000 | 0.020 | 0.012 | 0.017 | 0.000 | 0.010 | 0.013 | 0.027 | 0.023 | 1.353 | 0.923 |
| Coumarin | 0.000 | 0.000 | 0.000 | 0.005 | 0.000 | 0.002 | 0.000 | 0.000 | 0.000 | - | - |
| Cyclobutanone, 2,2-dimethyl- | 0.000 | 0.007 | 0.000 | 0.000 | 0.000 | 0.000 | 0.000 | 0.000 | 0.000 | - | - |
| Cycloheptane, methyl- | 0.006 | 0.000 | 0.003 | 0.005 | 0.001 | 0.000 | 0.000 | 0.005 | 0.003 | 3.829 | 0.372 |
| Cyclohexane, 1-ethyl-2,3-dimethyl- | 0.013 | 0.000 | 0.000 | 0.008 | 0.000 | 0.000 | 0.000 | 0.000 | 0.000 | - | - |
| Cyclohexene, 3,3,5-trimethyl- | 0.000 | 0.000 | 0.010 | 0.000 | 0.000 | 0.000 | 0.000 | 0.000 | 0.000 | - | - |
| Cyclooctane, methyl- | 0.004 | 0.004 | 0.003 | 0.000 | 0.000 | 0.000 | 0.000 | 0.000 | 0.000 | 6.401 | 0.059 |
| Cyclopentanecarbonitrile, 3-methylene- | 0.008 | 0.000 | 0.000 | 0.000 | 0.010 | 0.004 | 0.009 | 0.007 | 0.003 | 0.074 | **0.002** |
| Decanal | 0.030 | 0.000 | 0.021 | 0.000 | 0.000 | 0.000 | 0.000 | 0.007 | 0.000 | - | - |
| Diethyl carbitol | 0.000 | 0.031 | 0.022 | 0.046 | 0.000 | 0.009 | 0.015 | 0.000 | 0.032 | 3.128 | 0.517 |
| Dimethly sulfide | 0.035 | 0.003 | 0.000 | 0.000 | 0.000 | 0.003 | 0.006 | 0.067 | 0.000 | 0.070 | 0.228 |
| Disulfide, dimethyl | 0.035 | 0.000 | 0.000 | 0.000 | 0.007 | 0.012 | 0.005 | 0.000 | 0.005 | 0.098 | 0.064 |
| Dodecanoic acid, 1-methylethyl ester | 0.050 | 0.322 | 0.189 | 0.000 | 0.067 | 0.242 | 0.215 | 0.018 | 0.041 | 1.180 | 0.923 |
| epiphotocitral A | 0.000 | 0.000 | 0.003 | 0.000 | 0.002 | 0.004 | 0.007 | 0.005 | 0.000 | 0.160 | 0.059 |
| Ethanol, 2-(2-butoxyethoxy)- | 0.070 | 0.324 | 0.263 | 0.372 | 0.236 | 0.133 | 0.062 | 0.097 | 0.000 | 2.576 | 0.272 |
| Ethanol, 2-butoxy- | 0.021 | 0.052 | 0.006 | 0.000 | 0.046 | 0.012 | 0.034 | 0.023 | 0.000 | 0.663 | 0.892 |
| Ethanone, 1-(1-cyclohexen-1-yl)- | 0.000 | 0.000 | 0.000 | 0.003 | 0.000 | 0.000 | 0.000 | 0.000 | 0.000 | - | - |
| Ethanone, 1-(3,4-dimethoxyphenyl)- | 0.000 | 0.000 | 0.000 | 0.000 | 0.012 | 0.000 | 0.007 | 0.000 | 0.009 | 0.193 | 0.192 |
| Ethanone, 1-(3-methylphenyl)- | 0.000 | 0.009 | 0.004 | 0.000 | 0.000 | 0.000 | 0.000 | 0.003 | 0.000 | - | - |
| Ethanone, 1-(4-methylphenyl)- | 0.013 | 0.040 | 0.014 | 0.000 | 0.019 | 0.010 | 0.018 | 0.009 | 0.009 | 1.168 | 0.947 |
| Ethanone, 2,2'-(octahydro-2,3-quinoxalinediylidene)bis[1-phenyl- | 0.000 | 0.007 | 0.000 | 0.000 | 0.000 | 0.000 | 0.000 | 0.000 | 0.010 | - | - |
| ethene, 1,1'-[oxybis(methylenesulfonyl)]bis- | 0.007 | 0.013 | 0.000 | 0.000 | 0.000 | 0.008 | 0.000 | 0.003 | 0.000 | - | - |
| Ethoxyacetylene | 0.003 | 0.000 | 0.003 | 0.000 | 0.000 | 0.001 | 0.000 | 0.003 | 0.001 | 0.016 | 0.086 |
| Ethylbenzene | 0.001 | 0.010 | 0.002 | 0.006 | 0.000 | 0.001 | 0.001 | 0.018 | 0.000 | 2.114 | 0.254 |
| Eucalyptol | 0.000 | 0.000 | 0.000 | 0.038 | 0.154 | 0.115 | 0.014 | 0.000 | 2.194 | 0.007 | 0.106 |
| Furan, 2-methyl- | 0.000 | 0.000 | 0.002 | 0.007 | 0.000 | 0.000 | 0.000 | 0.000 | 0.001 | - | - |
| Heneicosane | 0.921 | 0.047 | 0.000 | 0.380 | 0.017 | 0.788 | 0.745 | 0.708 | 0.324 | 0.924 | 0.678 |
| Hexadecane | 0.000 | 0.000 | 0.000 | 0.000 | 0.000 | 0.000 | 0.051 | 0.000 | 0.000 | - | - |
| Homosalate | 0.006 | 0.000 | 0.005 | 0.000 | 0.000 | 0.000 | 0.000 | 0.002 | 0.000 | 82.968 | 0.102 |
| Hydrazine, ethyl- | 0.000 | 0.000 | 0.050 | 0.312 | 0.000 | 0.000 | 0.186 | 2.901 | 0.061 | 0.025 | 0.245 |
| Isobutane | 0.000 | 0.000 | 0.000 | 0.000 | 0.000 | 0.000 | 0.000 | 0.000 | 0.002 | - | - |
| Isoprene | 0.000 | 0.000 | 0.004 | 0.004 | 0.000 | 0.004 | 0.003 | 0.003 | 0.004 | 0.136 | 0.059 |
| Isopropyl Alcohol | 0.000 | 0.000 | 0.017 | 0.000 | 0.000 | 0.000 | 0.000 | 0.169 | 0.055 | - | - |
| Isopropyl palmitate | 0.002 | 0.000 | 0.004 | 0.000 | 0.000 | 0.001 | 0.003 | 0.004 | 0.001 | 0.057 | 0.066 |
| Mesitylene | 0.000 | 0.005 | 0.000 | 0.004 | 0.000 | 0.002 | 0.001 | 0.003 | 0.000 | 0.219 | 0.182 |
| Methacrolein | 0.000 | 0.000 | 0.002 | 0.002 | 0.000 | 0.000 | 0.000 | 0.000 | 0.000 | - | - |
| Methane, bromodichloro- | 0.014 | 0.222 | 0.004 | 0.003 | 0.000 | 0.000 | 0.000 | 0.003 | 0.000 | 41.134 | **0.018** |
| Methane, dibromo- | 0.000 | 0.000 | 0.000 | 0.002 | 0.000 | 0.000 | 0.003 | 0.002 | 0.000 | - | - |
| Methane, tribromo- | 0.000 | 0.000 | 0.000 | 0.000 | 0.000 | 0.000 | 0.000 | 0.000 | 0.000 | - | - |
| Methanesulfonic anhydride | 0.007 | 0.026 | 0.023 | 0.006 | 0.040 | 0.000 | 0.027 | 0.018 | 0.048 | 0.706 | 0.983 |
| Methyl methacrylate | 0.329 | 0.287 | 0.189 | 0.000 | 0.000 | 0.020 | 0.000 | 0.005 | 0.000 | 6.539 | 0.059 |
| Methyl α-methoxyphenylacetate | 0.006 | 0.000 | 0.000 | 0.000 | 0.006 | 0.003 | 0.004 | 0.002 | 0.000 | - | - |
| Methylene chloride | 0.039 | 0.042 | 0.008 | 0.008 | 0.029 | 0.012 | 0.080 | 0.060 | 0.000 | 0.733 | 0.983 |
| Naphthalene, 1,2-dimethyl- | 0.000 | 0.000 | 0.005 | 0.006 | 0.008 | 0.000 | 0.004 | 0.002 | 0.004 | 0.060 | 0.073 |
| Naphthalene, 1,4,6-trimethyl- | 0.000 | 0.000 | 0.000 | 0.000 | 0.002 | 0.002 | 0.000 | 0.000 | 0.000 | 0.017 | 0.235 |
| Napthalene | 0.000 | 0.000 | 0.000 | 0.005 | 0.000 | 0.000 | 0.000 | 0.000 | 0.007 | - | - |
| Nonanal | 0.066 | 0.053 | 0.015 | 0.001 | 0.000 | 0.000 | 0.000 | 0.030 | 0.000 | 87.854 | **0.004** |
| *Nonanoic acid* | 0.025 | 0.000 | 0.035 | 0.044 | 0.000 | 0.000 | 0.000 | 0.000 | 0.000 | 7.087 | 0.060 |
| Octanal | 0.005 | 0.016 | 0.005 | 0.000 | 0.000 | 0.001 | 0.000 | 0.004 | 0.000 | 5.569 | 0.059 |
| o-Cymene | 0.000 | 0.000 | 0.002 | 0.002 | 0.010 | 0.000 | 0.000 | 0.000 | 0.011 | - | - |
| Oxalic acid, heptyl propyl ester | 0.086 | 0.000 | 0.000 | 0.000 | 0.000 | 0.000 | 0.000 | 0.000 | 0.000 | - | - |
| Oxime-, methoxy-phenyl-_ | 0.000 | 0.000 | 0.000 | 0.000 | 0.109 | 0.058 | 0.000 | 0.000 | 0.026 | 0.072 | 0.208 |
| o-Xylene | 0.000 | 0.012 | 0.004 | 0.005 | 0.000 | 0.002 | 0.002 | 0.003 | 0.000 | 5.012 | 0.474 |
| Paroxypropione | 0.000 | 0.000 | 0.005 | 0.008 | 0.000 | 0.000 | 0.000 | 0.000 | 0.000 | - | - |
| Pentanal, 3-methyl- | 0.003 | 0.000 | 0.004 | 0.000 | 0.005 | 0.003 | 0.000 | 0.008 | 0.003 | 0.114 | 0.061 |
| Phenol | 0.008 | 0.000 | 0.009 | 0.033 | 0.008 | 0.000 | 0.000 | 0.008 | 0.017 | 3.203 | 0.569 |
| Phthalic acid, 4-fluoro-2-nitrophenyl methyl ester | 0.000 | 0.000 | 0.011 | 0.000 | 0.000 | 0.000 | 0.000 | 0.000 | 0.000 | - | - |
| Propanal, 2-methyl- | 0.039 | 0.000 | 0.000 | 0.000 | 0.000 | 0.029 | 0.036 | 0.000 | 0.006 | 0.054 | 0.209 |
| Propanoic acid, 2-hydroxyethyl ester | 0.016 | 0.103 | 0.037 | 0.074 | 0.060 | 0.027 | 0.047 | 0.000 | 0.048 | 1.564 | 0.570 |
| Propylene Glycol | 0.000 | 0.296 | 0.000 | 0.145 | 0.000 | 0.000 | 0.000 | 0.000 | 0.076 | - | - |
| Pyrimidine, 2,4-dimethyl- | 0.003 | 0.000 | 0.002 | 0.000 | 0.000 | 0.000 | 0.003 | 0.004 | 0.000 | - | - |
| Retene | 0.002 | 0.000 | 0.000 | 0.000 | 0.002 | 0.000 | 0.000 | 0.000 | 0.000 | - | - |
| Salicylic acid, tert.-butyl ester | 0.000 | 0.000 | 0.005 | 0.000 | 0.000 | 0.000 | 0.000 | 0.001 | 0.000 | - | - |
| Silane, triethylmethoxy- | 0.000 | 0.013 | 0.000 | 0.000 | 0.000 | 0.007 | 0.000 | 0.008 | 0.000 | - | - |
| Silanediol, dimethyl- | 0.000 | 0.239 | 0.000 | 0.050 | 0.000 | 0.000 | 0.000 | 0.035 | 0.000 | - | - |
| Silanol, trimethyl- | 0.000 | 0.000 | 0.052 | 0.134 | 0.000 | 0.000 | 0.000 | 0.167 | 0.236 | - | - |
| Sulfurous acid, 2-ethylhexyl hexyl ester | 0.029 | 0.000 | 0.010 | 0.000 | 0.020 | 0.000 | 0.029 | 0.025 | 0.009 | 0.085 | 0.064 |
| Sulfurous acid, 2-ethylhexyl isohexyl ester | 0.028 | 0.000 | 0.099 | 0.062 | 0.000 | 0.000 | 0.000 | 0.021 | 0.052 | 11.720 | 0.068 |
| Terpineol | 0.000 | 0.001 | 0.002 | 0.006 | 0.005 | 0.001 | 0.002 | 0.000 | 0.027 | 0.495 | 0.779 |
| Thiomorpholine | 0.000 | 0.017 | 0.017 | 0.000 | 0.015 | 0.000 | 0.000 | 0.012 | 0.011 | 0.164 | 0.192 |
| Toluene | 0.003 | 0.166 | 0.007 | 0.018 | 0.014 | 0.010 | 0.021 | 0.090 | 0.005 | 1.734 | 0.923 |
| Trichloromethane | 0.161 | 1.444 | 0.032 | 0.062 | 0.000 | 0.000 | 0.104 | 0.012 | 0.000 | 27.099 | **0.007** |
| Tricyclo[5.2.1.0(2,6)]dec-3-en-10-one | 0.004 | 0.002 | 0.001 | 0.001 | 0.007 | 0.002 | 0.002 | 0.002 | 0.001 | 0.684 | 0.983 |
| Tridecanoic acid, methyl ester | 0.000 | 0.000 | 0.000 | 0.006 | 0.000 | 0.000 | 0.000 | 0.000 | 0.000 | - | - |
| Trivinyl(chloromethyl)silane | 0.000 | 0.000 | 0.018 | 0.024 | 0.000 | 0.000 | 0.000 | 0.000 | 0.003 | - | - |
| Undecane | 0.012 | 0.000 | 0.010 | 0.000 | 0.014 | 0.000 | 0.019 | 0.000 | 0.000 | - | - |
| Unknown 100 | 0.001 | 0.002 | 0.001 | 0.002 | 0.004 | 0.003 | 0.003 | 0.011 | 0.002 | 0.540 | 0.272 |
| Unknown 110 | 0.002 | 0.003 | 0.007 | 0.012 | 0.000 | 0.000 | 0.000 | 0.000 | 0.000 | 27.297 | **0.004** |
| Unknown 111 | 0.003 | 0.006 | 0.002 | 0.000 | 0.000 | 0.001 | 0.003 | 0.002 | 0.000 | 2.186 | 0.759 |
| Unknown 112 | 0.005 | 0.004 | 0.001 | 0.000 | 0.002 | 0.002 | 0.003 | 0.004 | 0.000 | 1.177 | 0.883 |
| Unknown 117 | 0.000 | 0.000 | 0.000 | 0.003 | 0.006 | 0.000 | 0.002 | 0.000 | 0.004 | 0.010 | 0.095 |
| Unknown 118 | 0.001 | 0.000 | 0.001 | 0.000 | 0.003 | 0.002 | 0.002 | 0.002 | 0.002 | 0.077 | **0.001** |
| Unknown 121 | 0.004 | 0.000 | 0.000 | 0.000 | 0.000 | 0.000 | 0.002 | 0.001 | 0.000 | - | - |
| Unknown 123 | 0.001 | 0.005 | 0.001 | 0.000 | 0.005 | 0.004 | 0.002 | 0.001 | 0.000 | 0.579 | 0.923 |
| Unknown 125 | 0.000 | 0.000 | 0.000 | 0.000 | 0.001 | 0.001 | 0.000 | 0.001 | 0.000 | 0.209 | 0.182 |
| Unknown 126 | 0.002 | 0.000 | 0.000 | 0.000 | 0.000 | 0.000 | 0.000 | 0.000 | 0.000 | - | - |
| Unknown 128 | 0.002 | 0.003 | 0.001 | 0.001 | 0.004 | 0.000 | 0.002 | 0.000 | 0.000 | 9.537 | **0.000** |
| Unknown 130 | 0.000 | 0.000 | 0.000 | 0.002 | 0.001 | 0.001 | 0.000 | 0.001 | 0.000 | 1.125 | 0.983 |
| Unknown 131 | 0.001 | 0.001 | 0.000 | 0.001 | 0.003 | 0.001 | 0.003 | 0.001 | 0.002 | 0.532 | 0.474 |
| Unknown 135 | 0.000 | 0.000 | 0.000 | 0.000 | 0.065 | 0.000 | 0.000 | 0.000 | 0.000 | - | - |
| Unknown 136 | 0.000 | 0.000 | 0.013 | 0.000 | 0.006 | 0.000 | 0.000 | 0.000 | 0.000 | - | - |
| Unknown 154 | 0.000 | 0.000 | 0.004 | 0.000 | 0.000 | 0.000 | 0.000 | 0.000 | 0.000 | - | - |
| Unknown 157 | 0.000 | 0.000 | 0.000 | 0.000 | 0.003 | 0.000 | 0.000 | 0.000 | 0.000 | - | - |
| Unknown 159 | 0.000 | 0.000 | 0.000 | 0.000 | 0.000 | 0.000 | 0.000 | 0.000 | 0.015 | - | - |
| Unknown 160 | 0.000 | 0.000 | 0.011 | 0.000 | 0.000 | 0.000 | 0.000 | 0.000 | 0.000 | - | - |
| Unknown 161 | 0.000 | 0.000 | 0.000 | 0.000 | 0.000 | 0.012 | 0.000 | 0.000 | 0.000 | - | - |
| Unknown 165 | 0.000 | 0.000 | 0.004 | 0.001 | 0.000 | 0.003 | 0.003 | 0.006 | 0.000 | 0.245 | 0.181 |
| Unknown 168 | 0.000 | 0.000 | 0.000 | 0.000 | 0.000 | 0.000 | 0.000 | 0.002 | 0.000 | - | - |
| Unknown 169 | 0.000 | 0.000 | 0.000 | 0.000 | 0.000 | 0.000 | 0.000 | 0.001 | 0.000 | - | - |
| Unknown 178 | 0.000 | 0.000 | 0.000 | 0.031 | 0.000 | 0.000 | 0.000 | 0.000 | 0.000 | - | - |
| Unknown 185 | 0.000 | 0.000 | 0.003 | 0.005 | 0.000 | 0.000 | 0.000 | 0.000 | 0.000 | - | - |
| Unknown 192 | 0.000 | 0.000 | 0.000 | 0.000 | 0.000 | 0.000 | 0.006 | 0.000 | 0.000 | - | - |
| Unknown 198 | 0.000 | 0.027 | 0.000 | 0.000 | 0.000 | 0.000 | 0.000 | 0.000 | 0.030 | - | - |
| Unknown 199 | 0.000 | 0.019 | 0.000 | 0.000 | 0.000 | 0.000 | 0.000 | 0.000 | 0.000 | - | - |
| Unknown 201 | 0.000 | 0.008 | 0.000 | 0.000 | 0.000 | 0.000 | 0.000 | 0.000 | 0.006 | - | - |
| Unknown 210 | 0.000 | 0.000 | 0.000 | 0.007 | 0.000 | 0.000 | 0.000 | 0.003 | 0.006 | - | - |
| Unknown 220 | 0.000 | 0.000 | 0.006 | 0.008 | 0.000 | 0.000 | 0.000 | 0.000 | 0.002 | - | - |
| Unknown 225 | 0.000 | 0.000 | 0.019 | 0.000 | 0.000 | 0.000 | 0.000 | 0.000 | 0.000 | - | - |
| Unknown 226 | 0.000 | 0.000 | 0.007 | 0.000 | 0.000 | 0.000 | 0.000 | 0.000 | 0.000 | - | - |
| Unknown 233 | 0.000 | 0.000 | 0.006 | 0.011 | 0.000 | 0.000 | 0.000 | 0.000 | 0.000 | - | - |
| Unknown 236 | 0.000 | 0.000 | 0.003 | 0.000 | 0.000 | 0.000 | 0.000 | 0.000 | 0.002 | - | - |
| Unknown 237 | 0.000 | 0.000 | 0.002 | 0.003 | 0.000 | 0.000 | 0.000 | 0.000 | 0.000 | - | - |
| Unknown 243 | 0.000 | 0.000 | 0.000 | 0.000 | 0.000 | 0.000 | 0.000 | 0.000 | 0.008 | - | - |
| Unknown 251 | 0.000 | 0.000 | 0.000 | 0.005 | 0.000 | 0.000 | 0.000 | 0.000 | 0.005 | - | - |
| Unknown 255 | 0.000 | 0.000 | 0.000 | 0.007 | 0.000 | 0.000 | 0.000 | 0.000 | 0.000 | - | - |
| Unknown 27 | 0.000 | 0.030 | 0.000 | 0.000 | 0.026 | 0.014 | 0.000 | 0.015 | 0.019 | 0.099 | 0.064 |
| Unknown 35 | 0.000 | 0.001 | 0.000 | 0.007 | 0.000 | 0.011 | 0.000 | 0.000 | 0.018 | - | - |
| Unknown 5 | 0.036 | 0.000 | 0.000 | 0.000 | 0.028 | 0.005 | 0.029 | 0.000 | 0.000 | 0.082 | 0.203 |
| Unknown 54 | 0.004 | 0.009 | 0.033 | 0.000 | 0.085 | 0.030 | 0.000 | 0.000 | 0.016 | 0.491 | 0.923 |
| Unknown 55 | 0.001 | 0.015 | 0.001 | 0.005 | 0.011 | 0.006 | 0.004 | 0.000 | 0.002 | 0.867 | 0.892 |
| Unknown 58 | 0.008 | 0.000 | 0.004 | 0.005 | 0.004 | 0.012 | 0.013 | 0.007 | 0.010 | 0.740 | 0.598 |
| Unknown 64 | 0.004 | 0.007 | 0.002 | 0.006 | 0.013 | 0.006 | 0.005 | 0.004 | 0.006 | 0.738 | 0.976 |
| Unknown 72 | 0.000 | 0.008 | 0.000 | 0.009 | 0.020 | 0.015 | 0.009 | 0.003 | 0.023 | 0.018 | **0.003** |
| Unknown 74 | 0.012 | 0.000 | 0.008 | 0.012 | 0.002 | 0.000 | 0.000 | 0.016 | 0.000 | 4.531 | 0.474 |
| Unknown 76 | 0.017 | 0.000 | 0.000 | 0.000 | 0.000 | 0.015 | 0.000 | 0.000 | 0.000 | - | - |
| Unknown 81 | 0.000 | 0.002 | 0.001 | 0.000 | 0.004 | 0.004 | 0.002 | 0.001 | 0.003 | 0.030 | **0.003** |
| Unknown 83 | 0.002 | 0.007 | 0.005 | 0.012 | 0.000 | 0.000 | 0.000 | 0.000 | 0.003 | 17.396 | **0.002** |
| Unknown 98 | 0.001 | 0.000 | 0.002 | 0.000 | 0.004 | 0.003 | 0.001 | 0.000 | 0.000 | 0.003 | 0.109 |
| Unknown 99 | 0.007 | 0.000 | 0.000 | 0.000 | 0.000 | 0.000 | 0.000 | 0.000 | 0.000 | - | - |
| Unkown 147 | 0.000 | 0.000 | 0.000 | 0.000 | 0.009 | 0.000 | 0.000 | 0.000 | 0.005 | - | - |
| Unkown 148 | 0.000 | 0.000 | 0.000 | 0.000 | 0.008 | 0.004 | 0.000 | 0.000 | 0.000 | - | - |
| Unkown 149 | 0.000 | 0.000 | 0.000 | 0.017 | 0.004 | 0.000 | 0.002 | 0.002 | 0.000 | 0.103 | 0.203 |
| Unkown 150 | 0.000 | 0.000 | 0.003 | 0.000 | 0.003 | 0.000 | 0.002 | 0.000 | 0.000 | - | - |
| Unkown 151 | 0.000 | 0.000 | 0.000 | 0.000 | 0.007 | 0.003 | 0.000 | 0.000 | 0.000 | - | - |
| Unkown 203 | 0.000 | 0.005 | 0.000 | 0.000 | 0.000 | 0.000 | 0.000 | 0.000 | 0.000 | - | - |
| Unkown 204 | 0.000 | 0.006 | 0.000 | 0.000 | 0.000 | 0.000 | 0.000 | 0.000 | 0.000 | - | - |
| Unkown 205 | 0.000 | 0.004 | 0.004 | 0.005 | 0.000 | 0.000 | 0.000 | 0.000 | 0.000 | 9.109 | 0.064 |
| Unkown 66 | 0.000 | 0.000 | 0.000 | 0.000 | 0.017 | 0.006 | 0.000 | 0.000 | 0.003 | 0.059 | 0.217 |

**Table S6. Relative abundance and fold-change of all identified metabolites in control versus treatment (high light) groups.** Where data passed all quality control and filtering steps, the fold change between control and treatment and FDR corrected p-values are included. Significant values are in bold.

| **Compound** | **Control** | | | | **Treatment** | | | | | **Fold change** | **FDR corrected p-value** |
| --- | --- | --- | --- | --- | --- | --- | --- | --- | --- | --- | --- |
| 3-Hydroxybutyric acid | 0.140 | 0.145 | 0.136 | 0.120 | 0.236 | 0.216 | 0.089 | 0.057 | 0.191 | 0.755 | 0.127 |
| 4-Hydroxybenzoic acid | 0.009 | 0.007 | 0.004 | 0.008 | 0.010 | 0.007 | 0.005 | 0.003 | 0.012 | 0.841 | 0.529 |
| 4-Hydroxyproline | 0.647 | 1.516 | 0.371 | 1.363 | 0.765 | 0.522 | 0.495 | 0.204 | 0.357 | 0.113 | **0.000** |
| Adenine | 11.317 | 9.185 | 8.781 | 9.904 | 11.797 | 9.977 | 4.853 | 4.377 | 12.375 | 0.954 | 0.630 |
| Adenosine | 0.148 | 0.343 | 0.038 | 0.069 | 0.250 | 0.079 | 0.278 | 0.055 | 0.124 | - | - |
| Adipic acid | 0.007 | 0.006 | 0.005 | 0.005 | 0.008 | 0.006 | 0.003 | 0.002 | 0.008 | 0.942 | 0.861 |
| Alpha-Lactose | 0.049 | 0.030 | 0.059 | 0.001 | 0.040 | 0.027 | 0.039 | 0.023 | 0.008 | - | - |
| Arachidonic acid | 1.401 | 0.710 | 1.295 | 0.730 | 0.567 | 1.223 | 0.358 | 0.280 | 1.154 | 1.265 | 0.595 |
| Ascorbic acid | 0.003 | 0.002 | 0.003 | 0.003 | 0.003 | 0.002 | 0.002 | 0.001 | 0.005 | 0.964 | 0.983 |
| Azelaic acid | 0.003 | 0.004 | 0.002 | 0.003 | 0.004 | 0.003 | 0.001 | 0.001 | 0.004 | 1.145 | 0.674 |
| Benzoic acid | 0.209 | 0.212 | 0.219 | 0.229 | 0.271 | 0.217 | 0.125 | 0.113 | 0.277 | 0.903 | 0.372 |
| Beta-Alanine | 0.064 | 0.067 | 0.032 | 0.054 | 0.048 | 0.029 | 0.064 | 0.024 | 0.025 | 7.116 | **0.000** |
| Beta-Glycerophosphoric acid | 0.037 | 0.027 | 0.003 | 0.051 | 0.021 | 0.029 | 0.002 | 0.000 | 0.043 | - | - |
| Capric acid | 12.029 | 11.877 | 6.265 | 11.169 | 13.268 | 11.454 | 6.276 | 3.577 | 10.892 | 0.952 | 0.821 |
| Caproic acid | 0.381 | 0.263 | 0.226 | 0.232 | 0.388 | 0.356 | 0.190 | 0.135 | 0.385 | 0.789 | 0.057 |
| Caprylic acid | 0.267 | 0.193 | 0.055 | 0.196 | 0.280 | 0.195 | 0.075 | 0.027 | 0.279 | 0.944 | 0.966 |
| Citric acid | 0.058 | 0.043 | 0.059 | 0.036 | 0.050 | 0.027 | 0.047 | 0.037 | 0.039 | 6.932 | **0.000** |
| Creatinine | 0.128 | 0.099 | 0.140 | 0.137 | 0.108 | 0.103 | 0.062 | 0.088 | 0.136 | 0.983 | 0.983 |
| Alpha-aminobutyric acid | 0.002 | 0.004 | 0.002 | 0.004 | 0.003 | 0.002 | 0.002 | 0.002 | 0.002 | 1.059 | 0.923 |
| Arabinose | 0.011 | 0.040 | 0.006 | 0.011 | 0.010 | 0.009 | 0.006 | 0.005 | 0.009 | 0.171 | **0.000** |
| Fructose | 0.046 | 0.021 | 0.055 | 0.022 | 0.046 | 0.036 | 0.030 | 0.027 | 0.035 | 0.142 | **0.000** |
| Fucose | 0.024 | 0.020 | 0.016 | 0.026 | 0.021 | 0.018 | 0.016 | 0.010 | 0.024 | 0.941 | 0.860 |
| Galactose | 1.562 | 0.679 | 1.088 | 0.983 | 1.013 | 0.925 | 0.671 | 0.737 | 1.327 | 0.888 | 0.764 |
| Glucose | 6.069 | 2.705 | 2.995 | 3.753 | 4.237 | 3.845 | 1.810 | 2.372 | 4.749 | 0.907 | 0.731 |
| Glucuronic acid | 0.032 | 0.030 | 0.021 | 0.034 | 0.035 | 0.024 | 0.022 | 0.025 | 0.033 | 0.780 | 0.502 |
| Dihydroxyacetone | 0.012 | 0.007 | 0.005 | 0.006 | 0.010 | 0.006 | 0.005 | 0.005 | 0.012 | 0.808 | 0.474 |
| Maltose | 0.118 | 0.044 | 0.088 | 0.017 | 0.131 | 0.021 | 0.083 | 0.127 | 0.058 | - | - |
| Mannose | 0.990 | 0.463 | 1.666 | 0.867 | 0.556 | 0.476 | 1.049 | 0.844 | 1.251 | - | - |
| Docosahexaenoic acid | 0.426 | 0.252 | 0.358 | 0.216 | 0.219 | 0.382 | 0.134 | 0.065 | 0.314 | 1.237 | 0.569 |
| Docosapentaenoic acid | 0.056 | 0.026 | 0.036 | 0.031 | 0.024 | 0.050 | 0.011 | 0.007 | 0.043 | 1.228 | 0.570 |
| Dodecanedioic acid | 0.005 | 0.003 | 0.003 | 0.005 | 0.004 | 0.004 | 0.001 | 0.000 | 0.006 | 6.656 | **0.000** |
| Dodecanoic acid | 0.345 | 0.276 | 0.267 | 0.312 | 0.311 | 0.288 | 0.239 | 0.118 | 0.363 | 0.923 | 0.764 |
| Dopamine | 0.248 | 0.323 | 0.004 | 0.113 | 0.312 | 0.188 | 0.152 | 0.009 | 0.067 | - | - |
| Ribose | 0.182 | 0.088 | 0.137 | 0.121 | 0.114 | 0.104 | 0.087 | 0.069 | 0.182 | 0.944 | 0.892 |
| D-Ribose 5-phosphate | 0.006 | 0.008 | 0.003 | 0.017 | 0.011 | 0.006 | 0.006 | 0.001 | 0.009 | 0.085 | **0.000** |
| Ribulose | 0.433 | 0.170 | 0.254 | 0.206 | 0.209 | 0.202 | 0.111 | 0.092 | 0.430 | 1.084 | 0.893 |
| Tagatose | 0.086 | 0.041 | 0.073 | 0.019 | 0.105 | 0.084 | 0.037 | 0.036 | 0.040 | 0.101 | **0.000** |
| Xylitol | 0.002 | 0.002 | 0.002 | 0.002 | 0.002 | 0.002 | 0.002 | 0.002 | 0.003 | 0.704 | 0.302 |
| Xylose | 0.095 | 0.050 | 0.122 | 0.088 | 0.086 | 0.072 | 0.083 | 0.086 | 0.114 | 0.743 | 0.571 |
| Xylulose | 0.693 | 0.272 | 0.415 | 0.331 | 0.331 | 0.325 | 0.203 | 0.157 | 0.695 | 1.051 | 0.952 |
| Eicosapentaenoic acid | 1.428 | 0.720 | 0.842 | 0.623 | 0.713 | 1.320 | 0.387 | 0.226 | 0.956 | 1.090 | 0.860 |
| Elaidic acid | 0.139 | 0.057 | 0.084 | 0.038 | 0.088 | 0.115 | 0.034 | 0.022 | 0.081 | 1.035 | 0.976 |
| Fumaric acid | 0.030 | 0.021 | 0.014 | 0.024 | 0.032 | 0.022 | 0.015 | 0.008 | 0.030 | 0.859 | 0.449 |
| Galactitol | 0.011 | 0.009 | 0.015 | 0.012 | 0.013 | 0.012 | 0.015 | 0.015 | 0.014 | 6.803 | **0.000** |
| Galacturonic acid | 0.009 | 0.009 | 0.013 | 0.008 | 0.010 | 0.006 | 0.012 | 0.011 | 0.006 | 6.501 | **0.000** |
| Gluconic acid | 0.017 | 0.011 | 0.009 | 0.008 | 0.016 | 0.009 | 0.006 | 0.005 | 0.008 | 1.085 | 0.821 |
| Glucosamine | 0.011 | 0.004 | 0.006 | 0.007 | 0.005 | 0.007 | 0.004 | 0.005 | 0.009 | 0.967 | 0.966 |
| Glucose 6-phosphate | 0.003 | 0.003 | 0.001 | 0.006 | 0.005 | 0.002 | 0.002 | 0.000 | 0.003 | 0.056 | **0.001** |
| Glutaric acid | 0.015 | 0.011 | 0.011 | 0.008 | 0.014 | 0.011 | 0.008 | 0.008 | 0.017 | 0.770 | 0.241 |
| Glyceric acid | 0.503 | 0.326 | 0.327 | 0.326 | 0.372 | 0.226 | 0.192 | 0.161 | 0.428 | 1.089 | 0.717 |
| Glycerol | 0.465 | 0.176 | 0.478 | 0.166 | 0.267 | 0.285 | 0.237 | 0.167 | 0.321 | 0.142 | **0.000** |
| Glycerol 3-phosphate | 0.094 | 0.068 | 0.013 | 0.097 | 0.066 | 0.076 | 0.006 | 0.001 | 0.085 | - | - |
| Glycine | 8.130 | 10.449 | 8.898 | 9.695 | 11.915 | 8.695 | 6.341 | 5.128 | 9.624 | 0.900 | 0.569 |
| Glycolic acid | 11.968 | 10.696 | 4.112 | 11.346 | 14.112 | 10.261 | 3.936 | 0.910 | 14.972 | 9.564 | **0.000** |
| Guanine | 0.119 | 0.065 | 0.042 | 0.222 | 0.096 | 0.165 | 0.027 | 0.034 | 0.162 | 0.094 | **0.000** |
| Heptadecanoic acid | 0.036 | 0.027 | 0.025 | 0.027 | 0.040 | 0.032 | 0.019 | 0.009 | 0.055 | 0.817 | 0.569 |
| Hydroxyphenyllactic acid | 0.130 | 0.087 | 0.047 | 0.098 | 0.153 | 0.120 | 0.001 | 0.026 | 0.102 | 7.924 | **0.000** |
| Hydroxypropionic acid | 0.067 | 0.068 | 0.058 | 0.066 | 0.081 | 0.061 | 0.051 | 0.044 | 0.088 | 0.782 | 0.189 |
| Hypoxanthine | 0.026 | 0.013 | 0.008 | 0.016 | 0.026 | 0.017 | 0.007 | 0.005 | 0.013 | 1.029 | 0.979 |
| Indoleacetic acid | 0.004 | 0.002 | 0.007 | 0.002 | 0.003 | 0.002 | 0.001 | 0.002 | 0.008 | - | - |
| Itaconic acid | 0.006 | 0.005 | 0.004 | 0.007 | 0.006 | 0.004 | 0.002 | 0.002 | 0.006 | 1.087 | 0.674 |
| Alanine | 6.045 | 6.572 | 6.582 | 6.988 | 6.912 | 5.378 | 4.830 | 3.492 | 3.864 | 1.024 | 0.892 |
| Asparagine | 0.147 | 0.089 | 0.029 | 0.056 | 0.119 | 0.053 | 0.024 | 0.023 | 0.074 | 0.137 | **0.000** |
| Aspartic acid | 2.130 | 1.225 | 1.276 | 1.222 | 1.926 | 1.201 | 0.033 | 0.014 | 1.143 | 6.004 | **0.000** |
| Lathosterol | 0.005 | 0.006 | 0.006 | 0.004 | 0.005 | 0.004 | 0.005 | 0.002 | 0.008 | 0.870 | 0.779 |
| Cystathionine | 0.001 | 0.001 | 0.000 | 0.001 | 0.001 | 0.001 | 0.000 | 0.000 | 0.001 | - | - |
| Cysteine | 0.640 | 0.319 | 0.660 | 0.279 | 0.622 | 0.389 | 0.282 | 0.428 | 0.362 | 0.857 | 0.806 |
| Erythrulose | 0.020 | 0.030 | 0.017 | 0.100 | 0.043 | 0.024 | 0.018 | 0.032 | 0.045 | 0.115 | **0.000** |
| Levoglucosan | 0.030 | 0.038 | 0.034 | 0.026 | 0.053 | 0.035 | 0.019 | 0.019 | 0.036 | 0.840 | 0.359 |
| Glutamic acid | 1.044 | 0.637 | 0.587 | 0.742 | 0.804 | 0.602 | 0.330 | 0.257 | 0.591 | 1.205 | 0.182 |
| Glutamine | 0.633 | 0.442 | 0.081 | 0.904 | 0.317 | 0.221 | 0.141 | 0.042 | 0.776 | - | - |
| Homoserine | 0.007 | 0.005 | 0.004 | 0.001 | 0.006 | 0.002 | 0.002 | 0.004 | 0.002 | 16.720 | **0.000** |
| Linoleic acid | 0.269 | 0.151 | 0.172 | 0.093 | 0.296 | 0.303 | 0.124 | 0.063 | 0.284 | 0.702 | 0.262 |
| Isoleucine | 0.969 | 2.814 | 0.840 | 3.457 | 2.519 | 1.005 | 0.877 | 1.334 | 1.115 | - | - |
| Kynurenine | 0.092 | 0.072 | 0.107 | 0.052 | 0.068 | 0.056 | 0.059 | 0.042 | 0.162 | 0.867 | 0.834 |
| Lactic acid | 0.317 | 0.283 | 0.266 | 0.267 | 0.260 | 0.287 | 0.193 | 0.142 | 0.262 | 0.974 | 0.966 |
| Lysine | 0.040 | 0.014 | 0.044 | 0.023 | 0.020 | 0.025 | 0.008 | 0.004 | 0.024 | 0.112 | **0.000** |
| Methionine | 0.001 | 0.002 | 0.003 | 0.002 | 0.002 | 0.001 | 0.001 | 0.001 | 0.001 | 1.489 | 0.420 |
| Proline | 2.976 | 1.957 | 3.342 | 1.389 | 2.235 | 1.801 | 1.856 | 1.579 | 1.357 | 1.008 | 0.983 |
| Serine | 2.504 | 1.961 | 2.469 | 2.139 | 2.396 | 1.775 | 2.130 | 1.263 | 1.350 | 0.948 | 0.983 |
| Sorbose | 0.053 | 0.024 | 0.044 | 0.013 | 0.066 | 0.053 | 0.018 | 0.020 | 0.024 | 0.103 | **0.000** |
| Threonine | 0.750 | 1.528 | 0.743 | 1.726 | 1.182 | 0.754 | 1.135 | 0.618 | 0.693 | 0.988 | 0.983 |
| Tryptophan | 0.003 | 0.001 | 0.004 | 0.002 | 0.003 | 0.001 | 0.002 | 0.003 | 0.003 | - | - |
| Tyrosine | 0.084 | 0.073 | 0.055 | 0.042 | 0.233 | 0.066 | 0.057 | 0.056 | 0.072 | 0.545 | 0.192 |
| Valine | 3.832 | 6.257 | 2.856 | 7.050 | 5.260 | 3.751 | 2.554 | 2.636 | 3.411 | 1.101 | 0.895 |
| Lyxose | 0.045 | 0.019 | 0.029 | 0.027 | 0.021 | 0.014 | 0.014 | 0.010 | 0.018 | 1.502 | 0.189 |
| Malic acid | 0.007 | 0.007 | 0.003 | 0.004 | 0.009 | 0.005 | 0.004 | 0.002 | 0.003 | 0.921 | 0.947 |
| Mannitol | 0.160 | 0.094 | 0.339 | 0.037 | 0.239 | 0.194 | 0.252 | 0.286 | 0.100 | - | - |
| Methylmalonic acid | 0.005 | 0.008 | 0.004 | 0.010 | 0.007 | 0.005 | 0.003 | 0.003 | 0.004 | 1.115 | 0.889 |
| Methylsuccinic acid | 0.002 | 0.002 | 0.002 | 0.002 | 0.002 | 0.002 | 0.001 | 0.001 | 0.002 | 1.036 | 0.923 |
| Metoprolol | 0.196 | 0.185 | 0.057 | 0.235 | 0.236 | 0.174 | 0.038 | 0.012 | 0.276 | 12.125 | **0.000** |
| myo-Inositol | 2.574 | 1.155 | 1.583 | 1.241 | 1.450 | 1.193 | 1.013 | 0.849 | 1.930 | 0.995 | 0.966 |
| Myristic acid | 1.101 | 0.713 | 1.551 | 0.595 | 0.969 | 1.112 | 0.975 | 0.582 | 1.370 | 0.133 | **0.000** |
| N6-Acetyl-L-lysine | 0.009 | 0.007 | 0.005 | 0.006 | 0.006 | 0.008 | 0.004 | 0.002 | 0.010 | 0.953 | 0.923 |
| Acetyl-D-glucosamine | 0.218 | 0.113 | 0.136 | 0.146 | 0.132 | 0.112 | 0.098 | 0.070 | 0.177 | 1.027 | 0.952 |
| Acetylgalactosamine | 0.387 | 0.185 | 0.237 | 0.216 | 0.229 | 0.188 | 0.156 | 0.125 | 0.307 | 1.007 | 0.986 |
| Acetylglutamine | 0.118 | 0.055 | 0.058 | 0.074 | 0.084 | 0.076 | 0.035 | 0.046 | 0.091 | 0.918 | 0.767 |
| N-Acetyl-L-aspartic acid | 0.004 | 0.002 | 0.001 | 0.002 | 0.003 | 0.003 | 0.002 | 0.001 | 0.004 | 0.694 | 0.140 |
| Acetylmannosamine | 0.378 | 0.322 | 0.078 | 0.147 | 0.442 | 0.323 | 0.053 | 0.027 | 0.471 | - | - |
| Niacinamide | 0.016 | 0.025 | 0.012 | 0.021 | 0.026 | 0.016 | 0.017 | 0.019 | 0.037 | 0.598 | 0.192 |
| Nicotinic acid | 1.313 | 1.310 | 1.701 | 1.305 | 1.462 | 1.311 | 0.674 | 0.730 | 1.263 | 1.061 | 0.892 |
| Octadecanol | 0.518 | 0.398 | 0.366 | 0.293 | 0.453 | 0.429 | 0.265 | 0.163 | 0.618 | 0.859 | 0.432 |
| Oleamide | 0.004 | 0.004 | 0.002 | 0.004 | 0.003 | 0.002 | 0.000 | 0.002 | 0.004 | 5.957 | **0.000** |
| Oleic acid | 0.130 | 0.053 | 0.077 | 0.034 | 0.079 | 0.106 | 0.031 | 0.020 | 0.076 | 0.133 | **0.000** |
| Ornithine | 0.011 | 0.145 | 0.003 | 0.044 | 0.155 | 0.113 | 0.000 | 0.000 | 0.165 | - | - |
| Oxalic acid | 0.029 | 0.023 | 0.016 | 0.026 | 0.028 | 0.020 | 0.015 | 0.010 | 0.039 | 0.868 | 0.522 |
| Oxoglutaric acid | 0.001 | 0.003 | 0.003 | 0.004 | 0.004 | 0.002 | 0.002 | 0.001 | 0.001 | 1.192 | 0.891 |
| Palmitic acid | 9.878 | 7.897 | 7.902 | 6.330 | 9.392 | 8.669 | 4.951 | 3.374 | 11.474 | 0.894 | 0.450 |
| Palmitoleic acid | 1.681 | 0.702 | 1.264 | 0.626 | 0.998 | 1.650 | 0.554 | 0.420 | 1.356 | 0.919 | 0.786 |
| Pantothenic acid | 0.104 | 0.105 | 0.079 | 0.181 | 0.149 | 0.124 | 0.057 | 0.050 | 0.146 | 0.949 | 0.774 |
| Pelargonic acid | 0.188 | 0.119 | 0.062 | 0.142 | 0.153 | 0.138 | 0.071 | 0.026 | 0.173 | 0.988 | 0.983 |
| Phenylacetic acid | 0.010 | 0.007 | 0.009 | 0.010 | 0.013 | 0.010 | 0.006 | 0.003 | 0.012 | 0.890 | 0.456 |
| Phenyllactic acid | 0.146 | 0.072 | 0.093 | 0.094 | 0.106 | 0.106 | 0.032 | 0.033 | 0.119 | 1.117 | 0.759 |
| Phosphoric acid | 2.246 | 3.617 | 2.568 | 7.282 | 4.453 | 3.322 | 1.040 | 0.643 | 4.129 | 0.105 | **0.000** |
| Pimelic acid | 0.002 | 0.001 | 0.001 | 0.001 | 0.002 | 0.001 | 0.001 | 0.001 | 0.002 | 0.849 | 0.517 |
| Putrescine | 0.399 | 0.331 | 0.109 | 0.198 | 0.358 | 0.263 | 0.076 | 0.053 | 0.244 | 1.164 | 0.834 |
| Pyrocatechol | 0.003 | 0.003 | 0.001 | 0.003 | 0.003 | 0.002 | 0.001 | 0.001 | 0.005 | 0.896 | 0.860 |
| Pyroglutamic acid | 10.150 | 9.820 | 8.009 | 8.891 | 11.885 | 8.853 | 5.119 | 4.017 | 6.147 | 1.044 | 0.821 |
| Pyruvic acid | 0.070 | 0.068 | 0.064 | 0.058 | 0.100 | 0.077 | 0.035 | 0.031 | 0.075 | 0.875 | 0.262 |
| Ribitol | 0.014 | 0.008 | 0.013 | 0.010 | 0.010 | 0.008 | 0.009 | 0.007 | 0.015 | 0.911 | 0.884 |
| Sarcosine | 1.290 | 0.764 | 0.890 | 1.275 | 1.319 | 0.882 | 0.885 | 0.482 | 1.452 | 0.849 | 0.522 |
| Sorbitol | 0.014 | 0.009 | 0.014 | 0.017 | 0.013 | 0.010 | 0.016 | 0.013 | 0.011 | 7.814 | **0.000** |
| Stearic acid | 3.587 | 2.857 | 3.746 | 2.097 | 3.483 | 3.236 | 1.933 | 0.894 | 5.354 | 0.917 | 0.860 |
| Stigmasterol | 0.009 | 0.009 | 0.001 | 0.002 | 0.041 | 0.023 | 0.015 | 0.002 | 0.028 | 0.038 | **0.001** |
| Succinic acid | 0.096 | 0.056 | 0.065 | 0.076 | 0.082 | 0.066 | 0.033 | 0.031 | 0.089 | 1.026 | 0.947 |
| Sucrose | 1.927 | 0.734 | 1.023 | 0.188 | 1.129 | 0.863 | 0.711 | 0.773 | 0.233 | - | - |
| Threonic acid | 0.758 | 0.362 | 0.817 | 0.257 | 0.728 | 0.463 | 0.351 | 0.542 | 0.412 | - | - |
| Thymidine | 0.017 | 0.006 | 0.007 | 0.002 | 0.013 | 0.008 | 0.002 | 0.002 | 0.006 | 0.114 | **0.000** |
| Thymine | 9.046 | 3.100 | 6.035 | 7.416 | 4.890 | 7.313 | 1.128 | 2.102 | 9.418 | 1.157 | 0.798 |
| Trehalose | 1.144 | 0.399 | 0.707 | 0.124 | 1.189 | 0.209 | 0.609 | 0.921 | 0.386 | - | - |
| Triethanolamine | 0.267 | 0.258 | 0.181 | 0.194 | 0.139 | 0.147 | 0.112 | 0.078 | 0.191 | 1.331 | 0.111 |
| Tryptamine | 0.091 | 0.058 | 0.541 | 0.073 | 0.083 | 0.091 | 0.058 | 0.102 | 0.164 | - | - |
| Tyramine | 0.026 | 8.283 | 0.004 | 0.006 | 0.006 | 5.614 | 0.002 | 0.011 | 0.005 | - | - |
| Uracil | 2.067 | 0.744 | 1.493 | 1.412 | 1.001 | 1.123 | 0.442 | 0.532 | 1.529 | 1.294 | 0.566 |
| Urea | 0.277 | 0.195 | 0.141 | 0.147 | 0.260 | 0.184 | 0.112 | 0.134 | 0.219 | 0.816 | 0.376 |
| Xanthine | 0.000 | 0.090 | 0.091 | 0.187 | 0.151 | 0.099 | 0.045 | 0.000 | 0.150 | - | - |
